# EhFP10: A FYVE family GEF interacts with myosin IB to regulate cytoskeletal dynamics during endocytosis in *Entamoeba histolytica*

**DOI:** 10.1371/journal.ppat.1007573

**Published:** 2019-02-19

**Authors:** Gunjan Gautam, Mohammad Sabir Ali, Alok Bhattacharya, Samudrala Gourinath

**Affiliations:** School of Life Sciences, Jawaharlal Nehru University, New Delhi, India; University of Virginia, UNITED STATES

## Abstract

Motility and phagocytosis are key processes that are involved in invasive amoebiasis disease caused by intestinal parasite *Entamoeba histolytica*. Previous studies have reported unconventional myosins to play significant role in membrane based motility as well as endocytic processes. *Eh*Myosin IB is the only unconventional myosin present in *E*. *histolytica*, is thought to be involved in both of these processes. Here, we report an interaction between the SH3 domain of *Eh*Myosin IB and c-terminal domain of *Eh*FP10, a Rho guanine nucleotide exchange factor. EhFP10 was found to be confined to *Entamoeba* species only, and to contain a c-terminal domain that binds and bundles actin filaments. *Eh*FP10 was observed to localize in the membrane ruffles, phagocytic and macropinocytic cups of *E*. *histolytica* trophozoites. It was also found in early pinosomes but not early phagosomes. A crystal structure of the c-terminal SH3 domain of *Eh*Myosin IB (*Eh*MySH3) in complex with an *Eh*FP10 peptide and co-localization studies established the interaction of *Eh*MySH3 with *Eh*FP10. This interaction was shown to lead to inhibition of actin bundling activity and to thereby regulate actin dynamics during endocytosis. We hypothesize that unique domain architecture of EhFP10 might be compensating the absence of Wasp and related proteins in *Entamoeba*, which are known partners of myosin SH3 domains in other eukaryotes. Our findings also highlights the role of actin bundling during endocytosis.

## Author summary

*Entamoeba histolyica* is a highly motile human pathogen which eats the blood cells and immune cells by phagocytosis during progression of Amoebiasis disease. *E*. *histolytica* infections are a major concern in the developing countries. Myosins are motor proteins that move over actin cytoskeleton to drive the cellular processes. Unconventional myosins are a type of myosin which are different from myosin present in muscles, and are involved in regulation of membrane dependent processes crucial for cellular movement and endocytosis. In contrast to other eukaryotes, *Entamoeba* has only one unconventional myosin, Myosin IB which shows more similarity with metazoan myosins rather than amoeboid myosins. Myosin IB has been shown to be involved in phagocytosis. The exact role played by Myosin IB in the phagocytic process is still not fully understood.

SH3 domain is present at the c-terminal tail of Myosin IB which has been found to interact with proteins that regulate the actin cytoskeleton in other organisms. In this work, we have identified EhFP10 as one of the interacting proteins of EhMyosin IB SH3 domain through a co-crystal structure and biophysical experiments. Our localisation studies demonstrated the involvement of EhFP10 in phagocytosis and pinocytosis. This is the first report of the involvement of a FYVE domain containing GEF in pinocytosis. We have also analysed that EhFP10 has a unique c-terminal domain not present in any other FYVE family GEFs in *Entamoeba* as well as in other organisms. Actin binding studies indicated that the c-terminal domain of EhFP10 binds to actin filaments and leads to formation of thicker actin bundles. Myosin IB interaction with EhFP10 inhibits the formation of actin bundles. Through our results, we could hypothesize that the presence of a unique GEF like EhFP10 could compensate for the absence of WASP proteins in *E*. *histolytica* which have been found to interact with the myosin I SH3 domain in other organisms and regulate actin dynamics during endocytosis. Our study reveals a rare interaction of a myosin with a GEF, which interact to regulate actin bundling. EhMyosin IB is different from other amoeboid myosins and lies between the metazoan and amoeboid myosins like human myosin IE. Hence, the findings have broader implication to completely understand the closure stage of a phagocytic and pinocytic cup.

## Introduction

*Entamoeba histolytica* is the causative agent of amoebiasis disease in humans, a major public health problem in developing countries. Amoebiasis is the third-leading cause of deaths resulting from parasitic infections [1, 2]. The ability of *E*. *histolytica* to phagocytose cells of the intestinal epithelia and the immune system is the major contributor to its pathogenesis [3, 4]. Phagocytosis is associated with intensive cytoskeletal remodeling, which involves actin filaments, several actin-binding proteins, and myosins.

Unconventional myosin I constitutes the largest class of the myosin superfamily [5] and has been associated with a variety of membrane-based motility processes such as pinocytosis, membrane ruffling, lamellipodia and filopodia as well as phagocytosis [6, 7], but the detailed mechanisms and pathways of the associations of myosin I with these processes remain to be studied. Even in simple eukaryotes like yeast and amoebazoa, myosin I exists in several isoforms, which carry out distinct functions [8–10] but the genome of *E*. *histolytica* has a single isoform of myosin I, namely EhMyosin IB [11].

EhMyosin IB has been observed to localize in phagocytic cups during erythrophagocytic processes [12]. *Entamoeba* cells overexpressing EhMyosin IB were found to display decreased phagocytosis due to a defect in the phagosome initiation process [13]. The SH3 domain of *S*. *cerevisae* myosin I (Myo3 and Myo5) has been shown to interact with WASP-related proteins Vrp1, Bee1p and Las17p and help in the recruitment of myosin I and Arp2/3 proteins at cortical actin nucleation sites [14]. In other amoebae such as *Dictyostelium* and *Acanthamoeba*, the SH3 domain of myosin I interacts with adapter protein like CARMIL and Acan125 respectively, that help to recruit and activate Arp2/3 [15]. Formation of multiprotein complexes via these interactions is essential for rearrangement of the actin cytoskeleton during endocytosis. WASP and other WASP-related proteins function downstream of several GTPases in FcϒR-mediated phagocytosis, leading to actin polymerization at the site of cup formation [16]. So far, only calcium binding proteins (CaBPs), *Eh*CaBP3 and *Eh*CaBP5 have been shown to interact with EhMyosin IB, and they do not interact with its SH3 domain [12]. Crystal structure of the EhMySH3 domain provided details about the ligand preferences for interaction, [17] but no interacting partner for this domain has yet been identified.

Phagocytosis has been found to be a key determinant of the virulence of *E*. *histolytica* facilitating colonization of gut and other vital organs during invasive amoebiasis. In a wide variety of eukaryotes, membrane phospholipids play a significant role in membrane trafficking, cytoskeletal rearrangements and cell surface receptor signaling [18]. Formation of the phospholipid PI3P, on the phagosome surface by PI3 kinases results in the recruitment of several PI3P-binding proteins including EEA1 and Hrs [19]. Inhibition of PI3P blocks phagosome maturation. PI3P is involved in endocytosis and phagocytosis in *E*. *histolytica* cells as well [20, 21]. FYVE domains are considered biosensors for PI3P sites on the membranes [22]. The FYVE zinc finger domain has been hypothesized to associate only with phagosomes and not with fluid-filled pinosomes [22]. Consistent with this hypothesis, EhFP4 which is a FYVE domain containing Rho guanine nucleotide exchange factor (Rho-GEF) in *E*. *histolytica*, was found to be involved in phagocytosis. Several Dbl homology GEF proteins like EhGEF1, EhGEF2 and EhGEF3 present in *E*. *histolytica* have been studied in detail and found to play an important role in phagocytosis, cell motility and migration, capping and chemoattractive response [23–27] while EhFP4 is the only FYVE family GEF studied so far. The genome of *E*. *histolytica* harbors 12 FYVE family Rho GTPase exchange factors (EhFPs), of which EhFP 5, 6, 7 and 10 are highly expressed [21].

EhFP10 is the longest of the 12 FYVE family GEFs (EhFPs) and its gene expression has been detected in cultured clinical isolates and trophozoites, as well as in cysts [28, 29]. In a transcriptome analysis, *Eh*FP10 showed 2.7-fold upregulated expression in virulent *E*. *histolytica* compared to that in non-virulent *Rahman* strain of *E*. *histolytica* [29]. The presence of the EhFP10 protein in *E*. *histolytica* lysate was confirmed in a proteomic study by Marion et al. [30].

In the current work, we have identified a novel interaction between the SH3 domain of unconventional EhMyosin IB and the c-terminal domain of EhFP10, and showed this interaction to regulate the phagocytic and pinocytic processes by affecting the cytoskeletal dynamics. The c-terminal domain of EhFP10 was found to regulate actin dynamics as reported for the APC basic domain and tau protein. Our results also suggested the participation of EhFP10 in pinocytosis along with phagocytosis in *E*. *histolytica*. So far, EhFP10 is the first FYVE domain containing GEF which has been found to be involved in pinocytosis. Moreover, the EhFP10 localization pattern makes it as a marker for distinguishing between pinosomes and phagosomes within *E*. *histolytica* cells. EhFP10 has a unique domain organization and no homologs across all genomes available in BLAST databases; i.e., it was found only in *Entamoeba* species. Hence, this unique interaction could be of great advantage to the pathogen and may be responsible for its high motility and rate of phagocytosis.

## Results

### Identification of *Eh*FP10 as a potential binding partner for the *Eh*MySH3 domain

The SH3 domain recognizes the PXXP motif in proteins. These motifs have been further classified into class I [(K/R)xxPxxP] and class II [xPxxPx(K/R)] types [31, 32]. To identify proteins that interact with the c-terminal SH3 domain of *Eh*Myosin IB, a proteomics data by Marion et al. was utilized [30]. The data was obtained from cells overexpressing myosin IB and undergoing active erythrophagocytosis [33]. The screening was done using “SH3 HUNTER” software [50] as described in Material and Methods. A FYVE family Rho-GEF, *Eh*FP10 (accession no. EAL46050.1) was predicted to harbor the maximum number of PxxP motifs (Table 1). Since no unconventional myosin I homologs have been reported to be involved directly or indirectly, in the regulation of Rho mediated cytoskeletal regulation, we proceeded further with *Eh*FP10 in our current study.

**Table 1:** List of peptides selected using SH3 HUNTER software. Sequences shown in bold are the ones similar to those recognized by yeast myosin I (Myo3 and Myo5). Underlined sequences were used for binding studies using Surface Plasmon Resonance and co-crystallization experiments.

Accession No.	Protein	Peptides	
EAL46050.1	RhoGEF-PH-FYVE (EhFP10)	726–731 **PPIPHR** 864–870 RPQPPTP 723–729 **KVAPPIP** 731–737 RSLPPKP 24–29 PQLPPK	585–590 **PKLPPK** 757–763 **KQNPFVP** 867–872 PPTPKR 642–647 **PTVPPK** 734–739 **PPKPLR**
EAL52122.1	p21-activated kinase	151–156 PSVPAP	
EAL49974.1	Rho guanine nucleotide exchange factor, putative	413–419 VAPPSLP 444–452 REPPELPQY 416–421 PSLPNI	
EAL51743.1	protein kinase 2	354–359 PWIPPV	
EAL46413.1	Rho family GTPase	108–113 PNVPII 140–148 NIVPIQPPQ	
EAL44666.2	gamma-adaptin	670–675 PINPTP	
EAL51552.1	phosphatidylinositol 3-kinase, putative	536–541 PLNPRV	
EAL50557.1	myotubularin	189–194 **PDLPST**	
EAL46989.1	protein kinase with WD repeats	329–334 PNVPKE	

In a previous study, *Eh*MySH3 was predicted to preferentially bind pseudo-symmetrical and class II ligands [17]. Hence, to confirm the type of ligand interactions preferred by the *Eh*MySH3 domain, we selected a few peptide sequences from *Eh*FP10. Chosen ligands belonged to class I (P4), class II (P1), and an overlapping pseudo-symmetrical sequence belonging to both classes (P2), as predicted by “SH3 HUNTER’ software. Along with these, two PXXP peptides; P3 and P5 from a Rho GTPase, were also chosen (Table 2). All five peptides were chemically synthesized (Biochain Inc.) without any modifications.

**Table 2:** Details of peptides used for for SPR binding studies and co-crystallization experiments. Selected peptides belonged to all ligand types: Class I [(K/R)xxPxxP], Class II [xPxxPx(K/R)] and PxxP. Binding affinity denotes K_D_ (equilibrium dissociation constant).

Peptide name	Peptides sequences	Ligand Type	Parent Proteins	Amino acid location	Binding affinity (mM)
P1	SLPPKPLR	Class II	EhFP10	732–739	0.4
P2	KVAPPIPHR	Class (I + II)	EhFP10	723–731	1.8
P3	NIVPIQPPQ	PxxP	Rho family GTPase	140–148	1.0
P4	RPQPPTP	Class I	EhFP10	864–870	-
P5	QHCPNVPII	PxxP	Rho family GTPase	105–113	0.7

**Fig 1:**
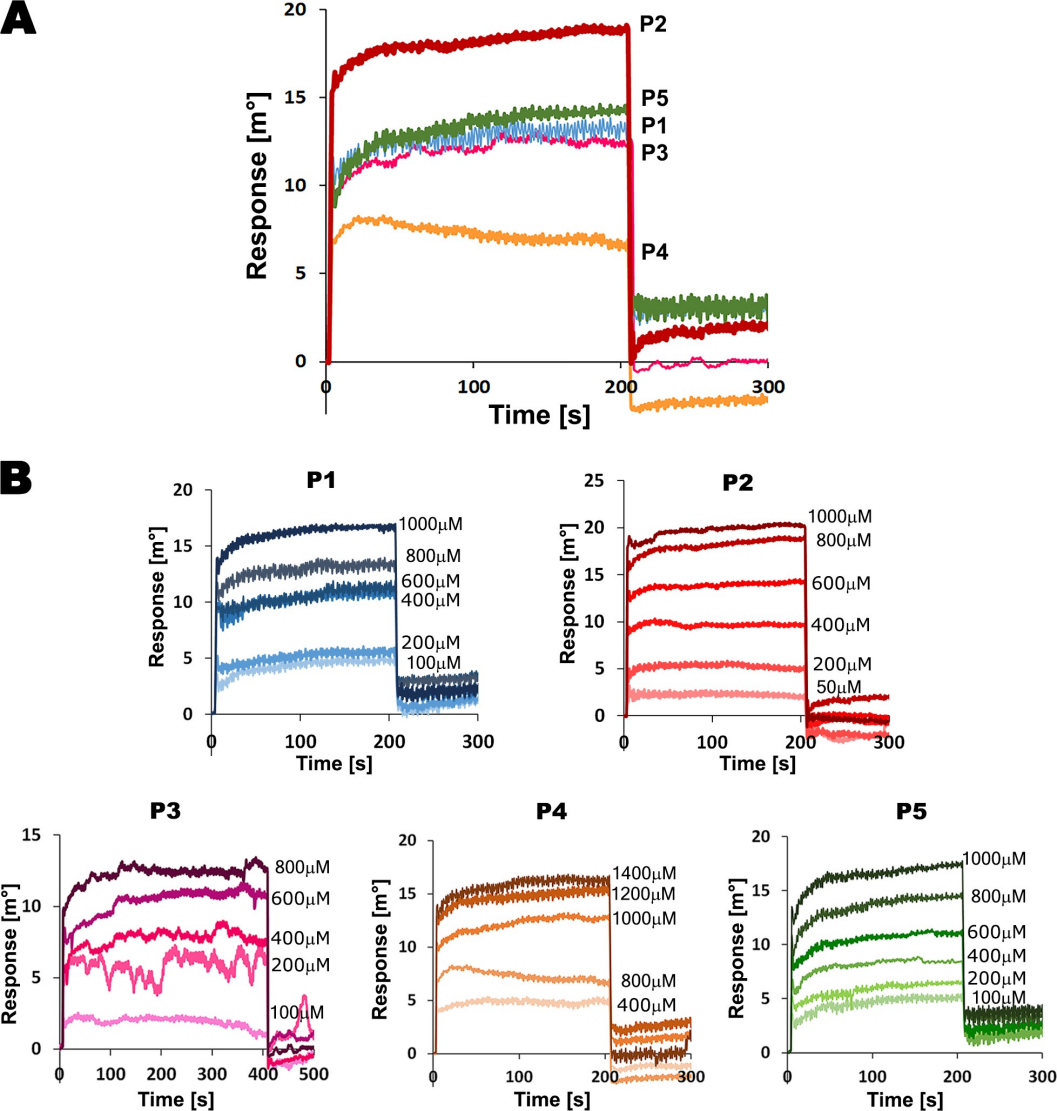
Interaction studies of EhMySH3 with peptides from EhFP10 and other proteins (Table 2) using Surface Plasmon Resonance. **(A)** SPR sensorgrams depicting binding responses when 0.8 mM of each peptide was passed over immobilized *Eh*MySH3 protein using Autolab SPR. (B) SPR kinetic analysis with each of the selected five peptides (P1, P2, P3, P4, and P5) over immobilized *Eh*MySH3 protein as a ligand.

These peptides were tested for their ability to bind EhMySH3 by using Autolab Surface Plasmon Resonance (SPR). The results showed that the P2 peptide, with a pseudosymmetrical ligand (overlapping class I and class II motif) from EhFP10 displayed highest binding response relative to other peptides, as was predicted in our previous study [17]. The weakest binding was observed with the P4 peptide (class I) (Fig 1). The binding affinity values of the peptides with the EhMySH3 domain could not be calculated accurately since the molecular mass of all of the peptides were about 1000 Da, close to the minimum detectable range for the Autolab instrument. Hence, on the basis of relative binding response, we proceeded with the high-response peptides, i.e., P2, P3, P1, and P5, for co-crystallization with EhMySH3.

### *Eh*MySH3 prefers the class II mode for interaction with pseudo-symmetrical P2 peptide from EhFP10

**Fig 2:**
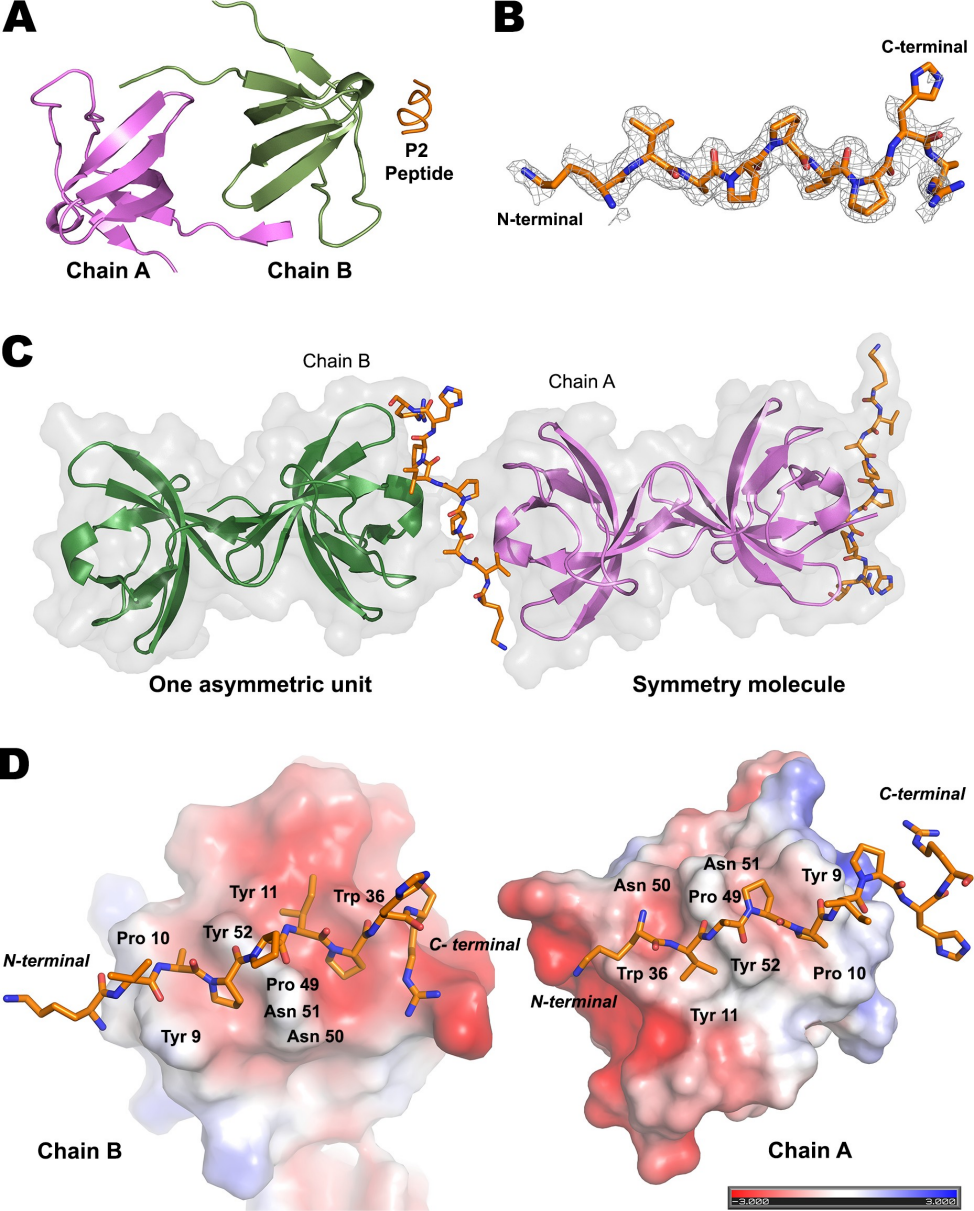
Crystal structure of EhFP10-P2 complex. **(A)** Crystal structure of the EhMySH3-P2 peptide complex, showing the asymmetric unit to be composed of two protein molecules, and one molecule of P2 near chain B. (B) 2Fo-Fc electron density map, of the P2 peptide of the EhMySH3-P2 complex at 1.5σ cut-off. (C) The structure of two asymmetric units of the crystal, showing one P2 peptide bound to two *Eh*MySH3 molecules. (D) Surface charge representation of the P2 peptide-binding site of chain B and chain A. The c-terminal Arg residue of P2 is bound in a class II orientation in the specificity pocket of chain B.

We were able to crystallize EhMySH3 in complex with the pseudo-symmetrical peptide P2 [KVAPPIPHR]. Co-crystallization attempts with other peptides resulted in the formation of the PEG-EhMySH3 complex instead of peptide-EhMySH3. The *Eh*MYSH3-P2 complex crystallized with two molecules of *Eh*MySH3 and one P2 peptide per asymmetric unit (Fig 2A), and with P2 binding to chain A of one *Eh*MySH3 molecule and chain B of a symmetry-related *Eh*MySH3 molecule (Fig 2C). P2 was observed to make more electrostatic interactions with chain B than with chain A. P2 binds to *Eh*MySH3 chain B in class II pattern, i.e., [xPxxPx(K/R)]. P2 residues A^3^ and P^4^ formed the first XP motif, which fit in the first consensus hydrophobic pocket made by residues Y^9^ and Y^52^ of *Eh*MySH3; and I^5^ and P^6^ formed the second XP motif, which occupied the second hydrophobic pocket made by residues P^49^ and W^36^ of *Eh*MySH3. The c-terminal residue R^9^ of P2 was observed to interact with residue E^34^ of the n-Src loop present in the specificity pocket of *Eh*MySH3. In other available ligand-bound SH3 structures, terminal Arg or Lys residues interact with a residue of the RT loop, which forms the specificity pocket. In our case, though the electron density for the terminal R^9^ residue was not complete, after refinement we obtained better electron density near the n-Src loop, though there could be an alternate conformation near E^18^ in the RT loop, likely to be one of the probable orientations for residue R^9^. Overall, we concluded that the *Eh*FP10 peptide, P2 binds to PxxP motif of *Eh*MySH3 in a class II peptide binding orientation and, due to its pseudo-symmetrical sequence, did so in a way resulting in the clustering of the two *Eh*MySH3 domains (Fig 2D).

### *Eh*Myosin IB interacts with the c-terminal domain of *Eh*FP10, a FYVE family Rho GEF

**Fig 3:**
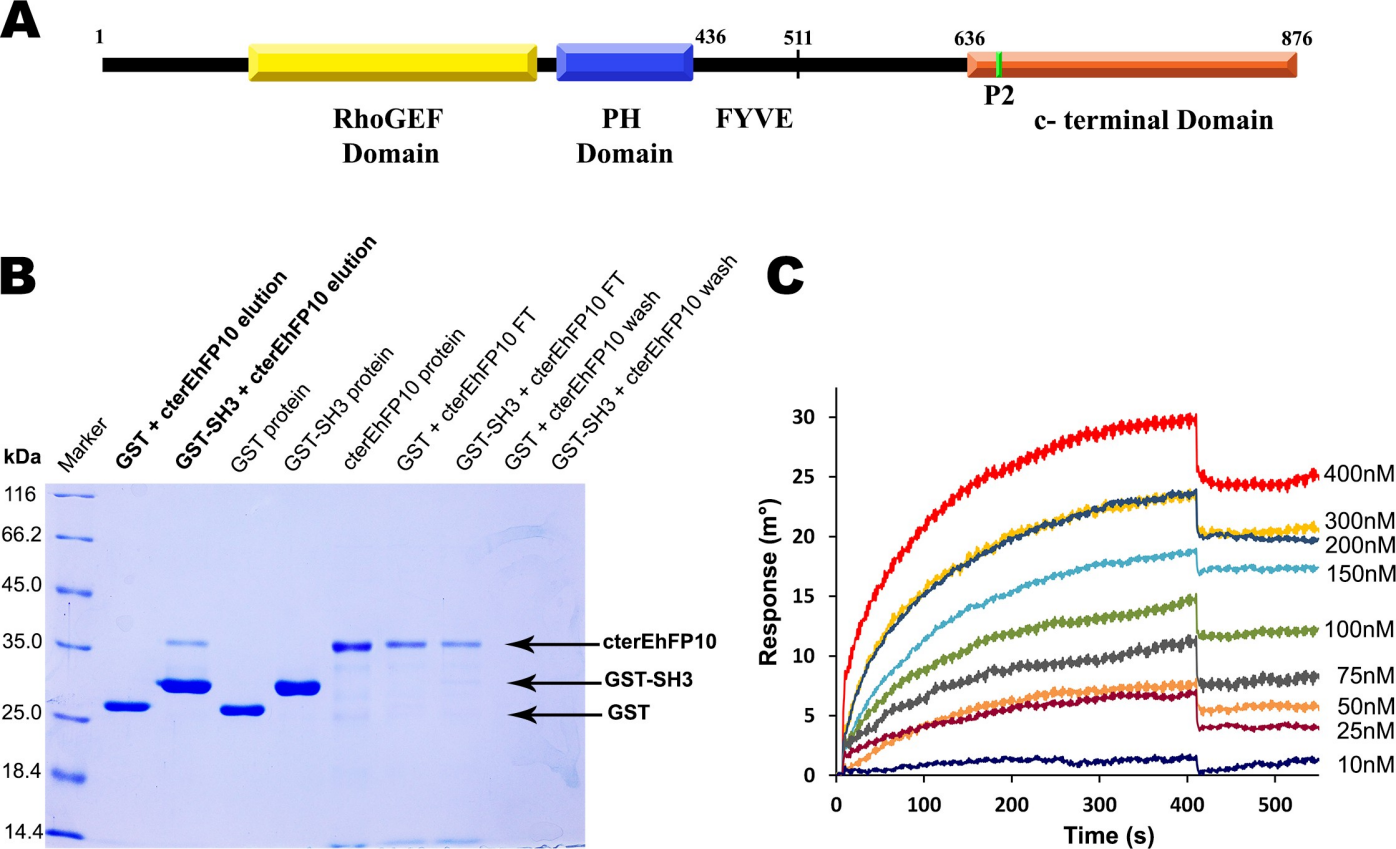
EhMySH3 interacts with c-terminal domain of EhFP10. **(A)** Schematic representation of EhFP10 protein showing RhoGEF, PH, FYVE, and c-terminal domains. The green line in the c-terminal domain marks the position of the peptide P2. (B) 12% SDS PAGE showing results of an in-vitro GST-pull down assay. ‘FT’ stands for flow through after binding, ‘Wash’ is the sample after using wash buffer and ‘Elution’ is the sample collected after using elution buffer, buffer composition is mentioned in materials and methods. cterEhFP10 was seen only in the GSTSH3+cterEhFP10 elution fraction, i.e., not in that of GST+cterEhFP10 elution. (C) SPR sensorgrams depicting results after cterEhFP10 protein samples of various concentrations were passed over immobilized EhMySH3 protein. The KD was calculated to be 200 nM.

EhFP10 is a multi-domain protein, 876 amino acids long, sub-divided into n-terminal Dbl homology GEF domain (1–438), followed by a FYVE domain (439–511) and a c-terminal domain (512–876) (Fig 3A). Based on sequence analysis and conserved domain database analysis, the c-terminal domain of EhFP10 (cterEhFP10) was predicted to be similar to the APC basic domain present in proteins of the adenomatous polyposis coli family and the microtubule-binding protein tau. Both APC basic and tau domains have been shown to bind microtubules as well as actin filaments [34]. They could also crosslink actin filaments and microtubules, in turn, mediate cytoskeleton crosstalk [35]. These observations and predictions suggest that the c-terminal domain of EhFP10 could associate with microtubules and actin filaments in *E*. *histolytica*.

On analysis of the domain organization of all 12 FYVE family GEFs present in *E*. *histolytica*, *Eh*FP10 was found to be the only GEF to have this unique c-terminal domain. The sequences of all Dbl-homology GEFs had highly conserved RhoGEF domain but different c-terminal domain. In a BLAST search across all databases, we could not find any GEF homologous to EhFP10. EhFP10 gene is present in all *Entamoeba* species. Homologues of EhFP10 of non- pathogenic strain *E*. *dispar* and *E*. *invadens* (pathogenic in reptiles), showed 95% and 35% identity with that of E. histolytica molecule respectively. Maximum differences were observed in the c-terminal domain (S3 Fig).

Peptide, P2 is located between 653–661 residues in the c-terminal domain of the *Eh*FP10 molecule (Fig 3A). To further confirm the *Eh*MySH3-EhFP10 interaction, an *in vitro* GST pull-down assay with recombinant cterEhFP10 protein and EhMySH3 was performed. Here, cterEhFP10 protein got eluted along with GST-tagged *Eh*MySH3 protein, and no cterEhFP10 protein was present with GST protein alone (Fig 3B). SPR studies were also carried out where various concentrations of cterEhFP10 protein samples were passed over immobilized *Eh*MySH3; these studies indicated a dissociation constant (KD) of 200 nm for *Eh*MySH3-cterEhFP10 (Fig 3C).

### *Eh*FP10 localizes in pseudopods during phagocytosis and pinocytosis in *E*. *histolytica* cells

*Eh*MyosinIB has been shown to be enriched in the extending pseudopods of motile amoeba and to also participate in the closure of the phagocytic cup by associating with phagocytic machinery [13]. Since *Eh*FP10 was found to be one of the interacting partners of *Eh*MyosinIB, we proceeded to study the cellular distribution of *Eh*FP10 in motile *E*. *histolytica* trophozoites. Localisation studies were carried out using cells expressing *Eh*FP10 tagged at its N terminus with GFP (GFP-*Eh*FP10). In immunostained cells, GFP-*Eh*FP10 was found mainly in the cytoplasm in proliferating cells. It was also seen in some membrane invaginations and cup-like projections (S4A Fig). The localisation showed a good correlation of GFP-EhFP10 with the untagged EhFP10 detected using EhFP10-specific antibody (S4B Fig).

**Fig 4:**
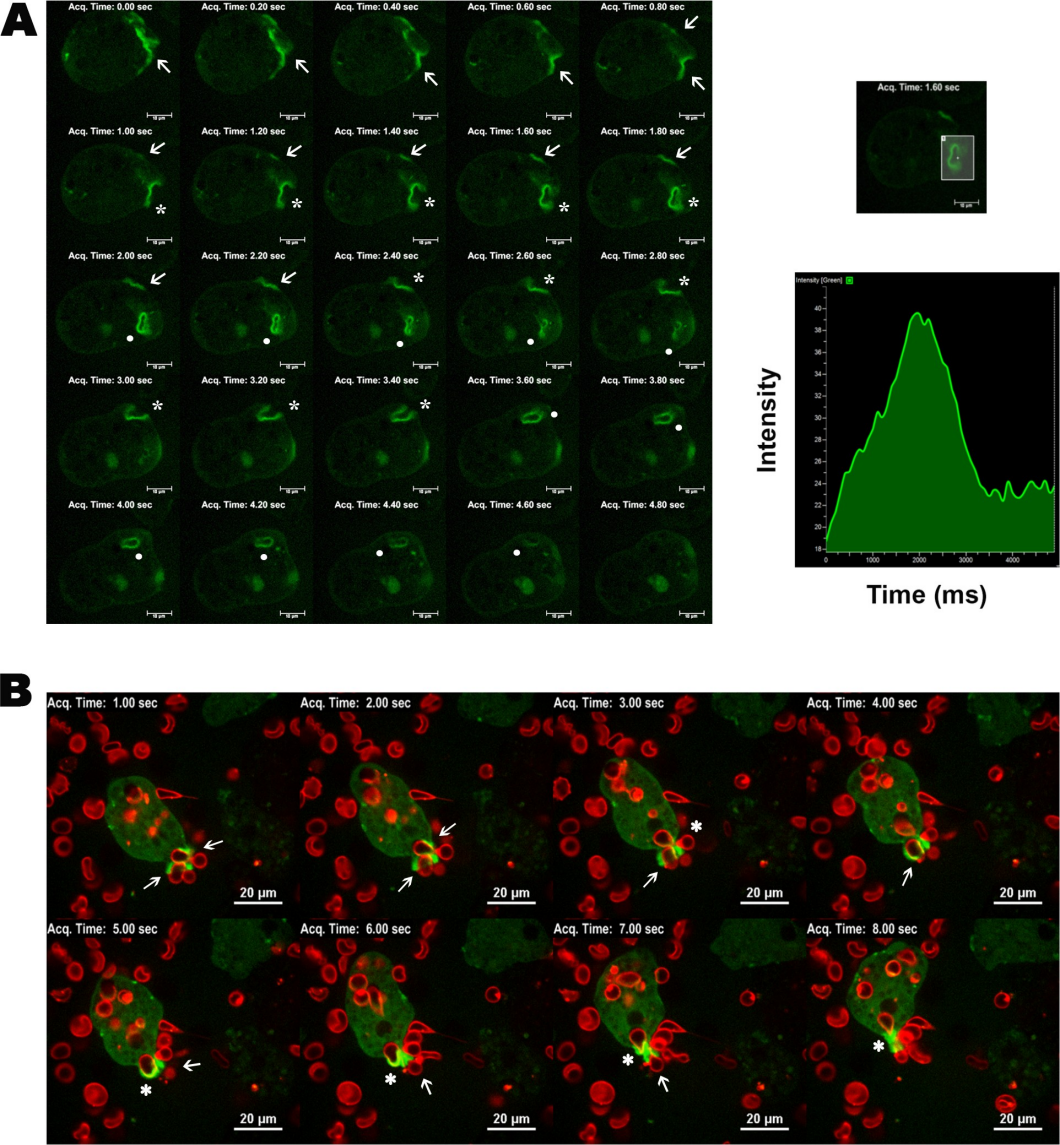
Localization of EhFP10 in *E*. *histolytica* cells showing its involvement in the phagocytic process. **(A)** Montage representing time series of images of GFP-EhFP10-expressing trophozoites under normal conditions. Several pseudopods leading to endocytic cup formation (marked with arrow), closing cups (marked with asterisks) and mature endosomes (marked with dots) were observed. (B) Montage representing time series of images of GFP-EhFP10-expressing trophozoites undergoing erythrophagocytosis. Enrichment of EhFP10 (bright green) in progressing pseudopods and in cups encircling RBCs (shown in red) leading to engulfment of these cells are marked with arrows. Closing phagocytic cups are marked with asterisks. Scale bar represents 10 μm.

To further look into the cellular distribution of EhFP10 in the dynamic cellular environment, time-lapse imaging was done using live proliferating amoebic trophozoites. GFP-EhFP10 was found to become enriched with time underneath the membrane at specific sites leading to ruffles and membrane protrusions. The level of enrichment initially increased with time at destined specific site near the membrane. Later the enrichment expanded along the membrane, and finally divided into two enriched sites leading to the simultaneous formation of two macropinocytic cups. The two cups then completed the macropinocytosis independently, usually one after the other. This pattern was recurrent and appeared to be the common mechanism of formation of the pinocytic vesicles. The GFP-*Eh*FP10 protein was found to be associated with the cup from initiation until scission and also in internalized macropinosomes (Fig 4A and S1 Video).

**Fig 5:**
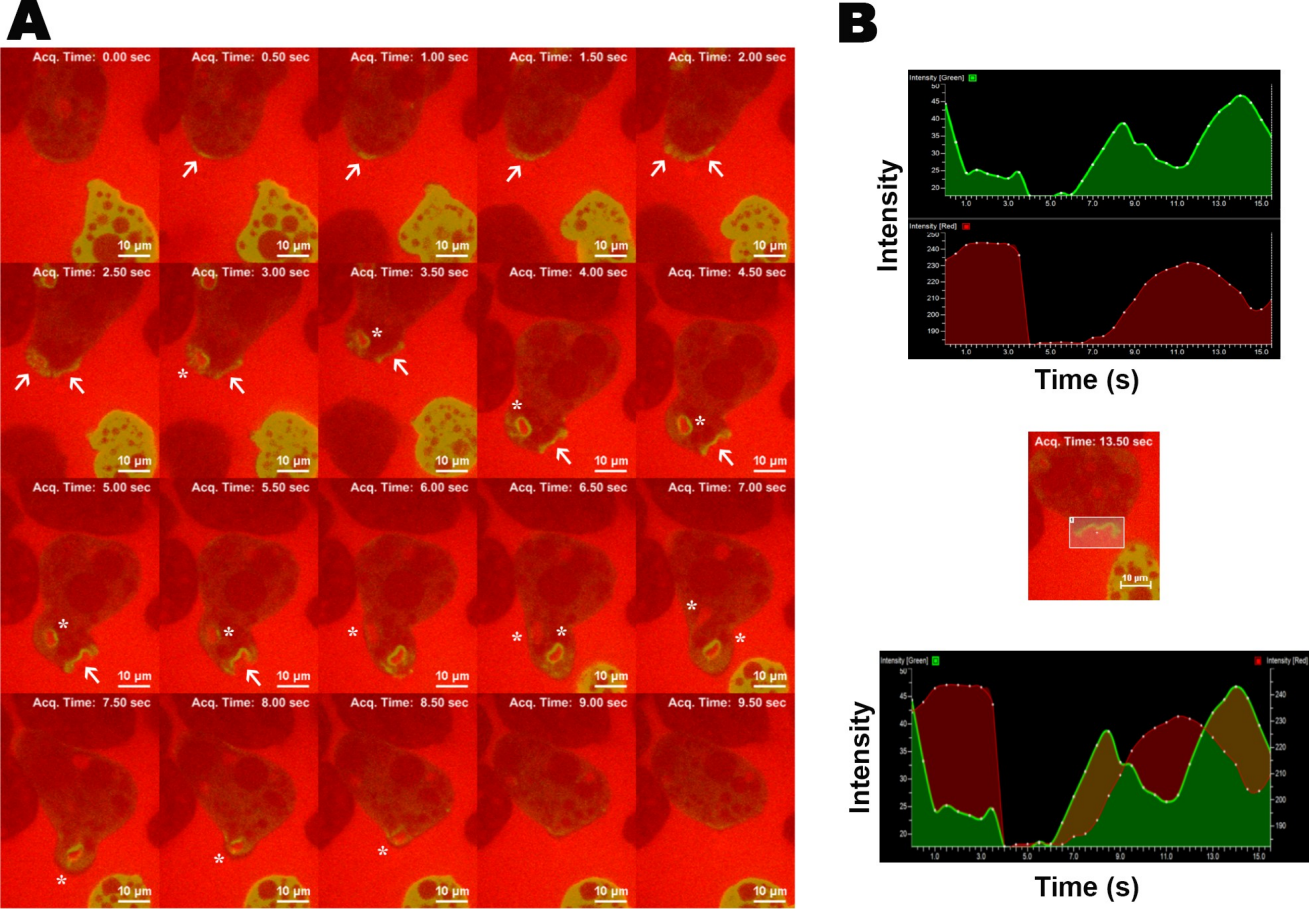
Localization of *Eh*FP10 in *E*. *histolytica* cells showing its involvement in the macropinocytic process. (A) A montage representing a time series (0 to 9.5 seconds) of images of GFP-*Eh*FP10-expressing trophozoites undergoing macropinocytosis from live cell microscopy. Enrichment of *Eh*FP10 in progressing pseudopods (marked with arrows) and engulfment of labeled media (red colour) within the vesicle (marked with asterisks) were observed. Scale bar represents 10 μm. (B) The intensity profile (ROI) of red (i.e. labelled media) and green fluorescence (i.e. GFP tagged EhFP10 protein) is shown as a function of time. Image at center depicts the snapshot of an *E*. *histolytica* trophozoite undergoing macropinocytosis. The rectangle marks the selected region that encloses a macropinocytic cup. An increase in the intensity of red colour from 9 s to 14 s was observed within the green region (i.e., amoeba cell), indicating the formation of a macropinocytic cup and presence of red fluorescence of media within the *E*. *histolytica* cell. Bottom image demonstrates overlapped red and green intensity profile in the marked region and top image displays individual profiles of each colour in the marked region.

To confirm the endocytic process to be pinocytosis, TRITC-dextran (Sigma) was added to the media, and endowed the media with red fluorescence. The presence of red fluorescence within internalized vesicles enriched with GFP-*Eh*FP10 confirmed *Eh*FP10 to be involved in fluid-phase macropinocytosis in *E*. *histolytica*. *Eh*FP10 was present from initiation until scission of the macropinocytic vesicles as well as around internalized macropinosomes. A single macropinocytic event typically took 4–5 seconds to complete. (Fig 5A and S2 Video).

Since, phagocytosis plays a crucial role in amoebiasis disease pathogenesis, we were interested to determine whether EhFP10 also plays a role in phagocytosis in addition to macropinocytosis. GFP-*Eh*FP10-overexpressing cells were utilized for this purpose. RBC membranes were stained with lipophilic, red fluorescent dye (Thermo Fisher). *Eh*FP10 was seen to be enriched at the site of pseudopod formation near the cell membrane as soon as RBC came in contact with *E*. *histolytica* cells, and it later became enriched in the extending pseudopods during the cup progression and was present until phagosome scission (Fig 4B). The distribution of GFP-EhFP10 in the phagocytic process, however, was different from that for pinocytosis after the scission of the early endosome. Unlike macropinosomes, the phagosome membrane was not enriched with EhFP10. An erythrophagocytic process took about 7–8 seconds to complete (Fig 4B and S3 Video).

To sum up, *Eh*FP10 was found enriched in membrane ruffles and around the sites of both macropinocytosis as well as phagocytosis. It was enriched in the pseudopods during cup progression and was present until scission of the vesicle. A single macropinocytic event transpired in a shorter amount of time than did a single phagocytic event. This is the first report of the involvement of a FYVE family RhoGEF of *E*. *histolytica* in macropinocytosis. Previously FYVE family GEFs were hypothesized to be associated only with phagocytosis [22]. The difference in the localization patterns of EhFP10 in phagocytosis and pinocytosis; and the presence of EhFP10 in the membranes of early macropinosomes but not in those of phagosomes, established this protein as a potential marker to distinguish pinosomes from phagosomes in *Entamoeba* species.

### *Eh*FP10 co-localizes with *Eh*Myosin IB and F-actin during phagocytosis and pinocytosis

**Fig 6:**
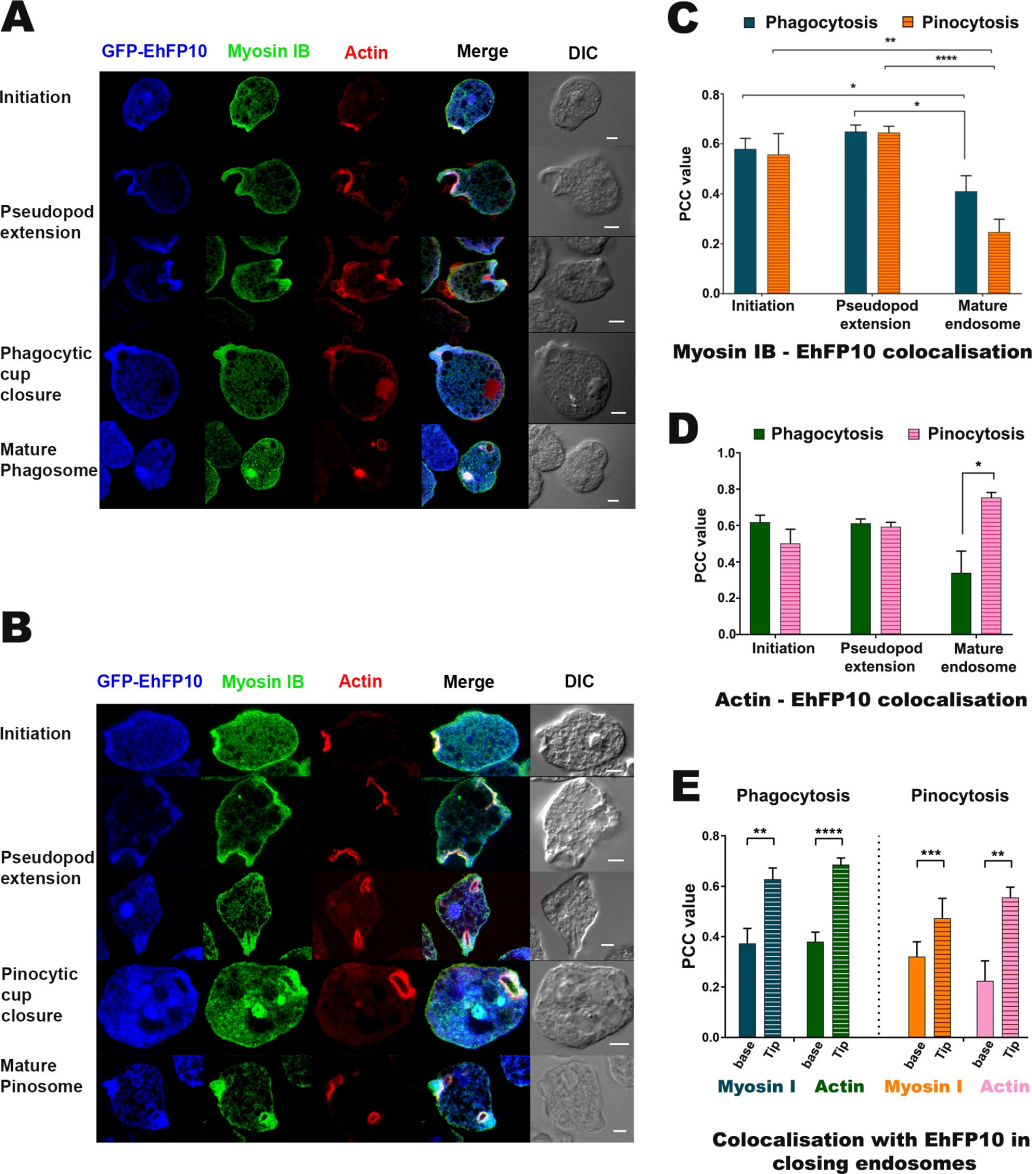
Colocalization of myosin IB with *Eh*FP10 during phagocytosis and pinocytosis. (A) Confocal images of immunostained fixed *E*. *histolytica* cells depicting colocalization of myosin IB with *Eh*FP10 at different stages of phagocytosis. (B) Immunostained fixed confocal images showing myosin IB to have also colocalized with *Eh*FP10 during pinocytosis from initiation to cup closure as well as in internalized pinosomes. Scale bar represents 5 μm. (C, D) Quantitative depictions of the colocalization of *Eh*FP10 with (C) *Eh*Myosin IB and with (D) actin, each at various stages of phagocytosis and pinocytosis. (E) Quantitative depiction of the colocalization of *Eh*FP10 with myosin IB and actin in the base and tip region of closing vesicles during phagocytosis and pinocytosis. Pearson’s correlation coefficient (PCC) was used to quantify colocalization. PCC values were calculated using Olympus Fluoview FV1000 software by selecting area enclosing the phagocytic and pinocytic cups, and also the area at the base and the tip of the endocytic cups from different trophozoites (n) and the mean values were plotted. Statistical analysis was performed using the unpaired two-tailed Student’s t-test (*P < 0.05, **P<0.01, ***P< 0.001, ****P< 0.0001 was considered significant). Error bar = SEM. *Eh*FP10-Myosin [Phagocytosis/ Pinocytosis (initiation, n = 19/ n = 7; Extension, n = 26/ n = 13; Phagosome, n = 5/ n = 13; Base, n = 9/ n = 12; Tip, n = 9/n = 12)]. *Eh*FP10-Actin [Phagocytosis/ Pinocytosis (initiation, n = 19/ n = 6; Extension, n = 26/ n = 15; Phagosome, n = 5/ n = 19; Base, n = 9/ n = 12; Tip, n = 9/n = 12)].

The SH3 domain of *Eh*Myosin IB was shown, as described above, to interact with the c-terminal domain of *Eh*FP10. This interaction was further validated by measuring co-localization in images of immunostained actively proliferating amoebic cells overexpressing GFP-*Eh*FP10. *Eh*Myosin IB was found to colocalize with *Eh*FP10 at the pinocytic cups as well as at the macropinosomes (Fig 6B and 6C). In cells undergoing phagocytosis, a similar association was observed between *Eh*FP10 and *Eh*Myosin IB (Fig 6A and 6D). Since phagocytosis and macropinocytosis are actin-dependent processes, and since homologs of the c-terminal domain of *Eh*FP10 are involved in actin assembly, we investigated the association of *Eh*FP10 with actin within *E*. *histolytica* cells.

Interestingly, in almost every cell where F-actin (stained with TRITC phalloidin) was observed, there was an enrichment of *Eh*FP10. The corresponding Pearson’s correlation coefficient was calculated to be between 0.5 and 0.6, suggesting a likely association of *Eh*FP10 with actin dynamics. However, in cells undergoing erythrophagocytosis, *Eh*FP10 and *Eh*Myosin IB colocalized at the proximal end (tip) of progressing pseudopods while actin was present both at the tip and the base of the cup. As the cups progressed towards closure, all three proteins were found to colocalize at the tip of the closing pinocytic and phagocytic vesicles (Fig 6E). As the pseudopods fused, *Eh*FP10 and *Eh*Myosin IB moved more towards the tip of the closing pseudopods in the case of phagocytosis; while during pinocytosis, *Eh*FP10 also resided at the membranes of closing pinosomes. In previous studies, myosin IB was reported to help in the pinching of the vesicle. On the basis of our localization studies, we suggest that *Eh*FP10 also plays a role in this process. Neither *Eh*FP10 nor *Eh*Myosin IB were present in the internalized phagocytic vesicles (Fig 6A). Images of immunostained fixed amoebic cells also confirmed the presence of *Eh*FP10 throughout the pinocytic process including in macropinosomes, in contrast to the case for phagocytosis where *Eh*FP10 was no longer observed during the separation of phagosomes from membranes. However, in early pinosomes, *Eh*FP10 was found to colocalize more with actin than with myosin IB (Fig 6C and 6D).

These observations clearly pointed out the difference in molecular mechanisms governing macropinocytosis and phagocytosis and the involvement of *Eh*FP10 and *Eh*Myosin IB in both of the processes.

### The C-terminal domain of *Eh*FP10 binds and bundles actin filaments like classical APC basic domain

**Fig 7:**
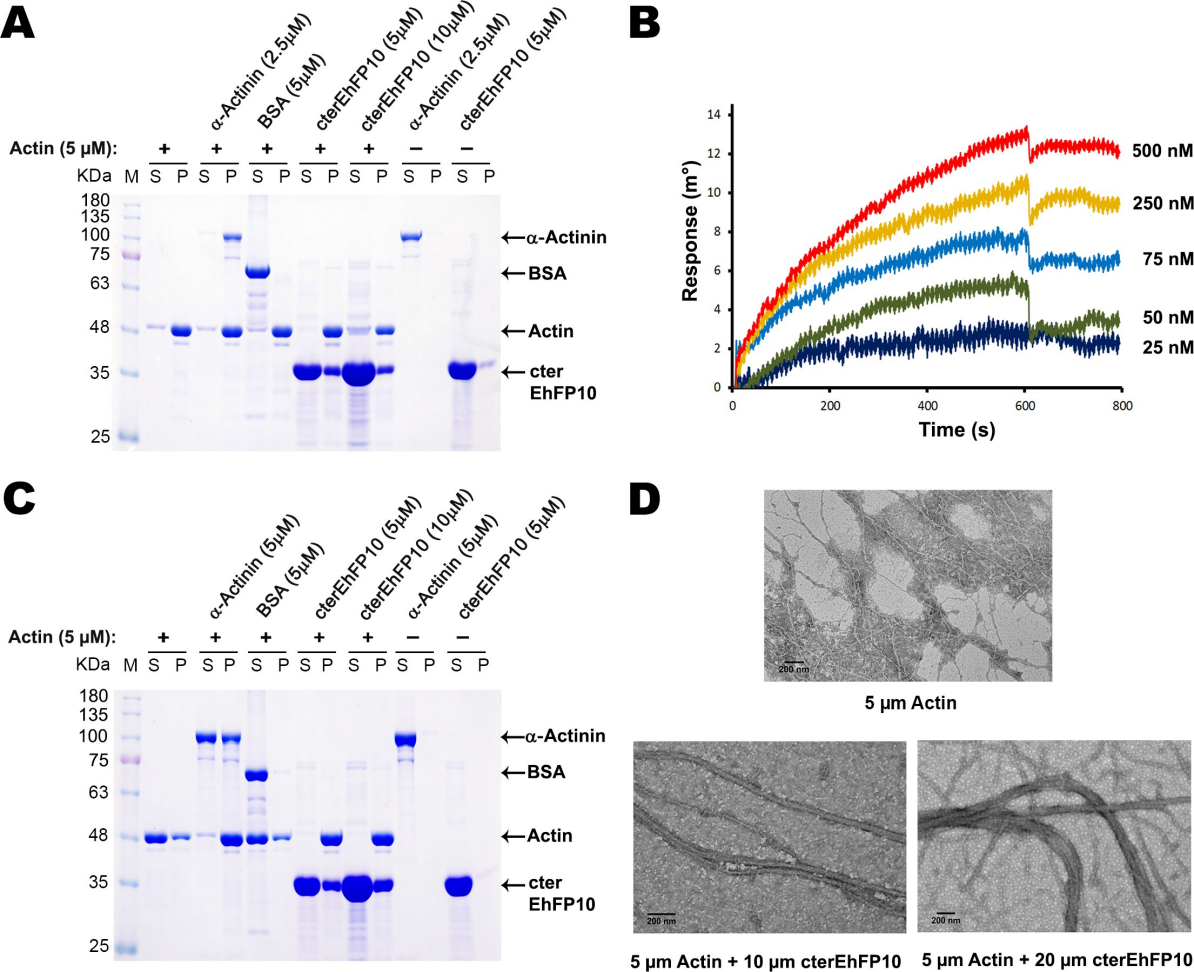
*Eh*FP10 binds and bundles actin filament. (A) 10% SDS-PAGE gel of the supernatant (S) and pellet (P) fractions obtained after co-sedimentation assay of purified filamentous rabbit muscle actin with purified cter*Eh*FP10. Purified cter*Eh*FP10 protein was seen to co-sediment with actin and was found in the pellet fraction. Purified Alpha-actinin (cytoskeleton Inc.) was used as positive control and bovine serum albumin (SRL) was used as a negative control in the experiment. (B) SPR sensorgrams obtained after cter*Eh*FP10 proteins of various concentrations were passed over immobilized F-actin. The coresponding KD value was determined to be 87 nM. (C) 10% SDS-PAGE gel of the supernatant (S) and pellet (P) fractions obtained after actin bundling assay of purified filamentous rabbit muscle actin with purified cter*Eh*FP10. cter*Eh*FP10 was found to bundle F-actin as only F-actin stayed in supernatant while heavier bundled actin went into the pellet fraction along with cter*Eh*FP10 upon low-speed centrifugation. Purified Alpha-actinin (cytoskeleton Inc.) was used as positive control and bovine serum albumin (SRL) was used as a negative control in the experiment. (D) TEM images showing changes in the thickness of F-actin as the cter*Eh*FP10 concentration was changed. Scale bar represents 100 nm.

APC basic and tau domains have been shown to be involved in actin dynamics [34]. Since *Eh*FP10 has a c-terminal domain that shows similarity with APC basic and tau domains, a direct involvement of *Eh*FP10 in actin dynamics was investigated. Purified recombinant cter*Eh*FP10 domain was observed to bind and bundle purified rabbit muscle F-actin in centrifugation-based actin binding (Fig 7A) and bundling assays (Fig 7C and 7D). In actin co-sedimentation assay at high-speed centrifugation of these proteins, a fraction of the cter*Eh*FP10 was seen in the pellet along with F-actin, confirming its interaction with F-actin, and only cter*Eh*FP10 (i.e., no actin) was observed in the supernatant (Fig 7A). The bundling assay was carried out using low-speed centrifugation. Here, in the absence of cter*Eh*FP10, F-actin stayed in the supernatant fraction, while in the presence of cter*Eh*FP10, almost all of the actin went into the pellet fraction (Fig 7C). However, in both cases, the amount of cter*Eh*FP10 that bound the actin remained almost constant for a fixed actin concentration. Bundles of F-actin were also visualized using TEM. As the concentration of the cter*Eh*FP10 protein was increased, thicker actin bundles were observed (Fig 7D); specifically, actin filaments, which were around 4 nm to 7 nm in thickness, grew to as thick as 50 nm to 120 nm in diameter in the presence of 10 μM to 20 μM cter*Eh*FP10 (Fig 7D). The KD of cter*Eh*FP10 for actin was calculated using SPR to be about 87 nM (Fig 7B), indicating a stronger affinity of *Eh*FP10 for actin than for *Eh*MySH3.

### *Eh*MySH3 binds *Eh*FP10 and inhibits its F-actin bundling activity

**Fig 8:**
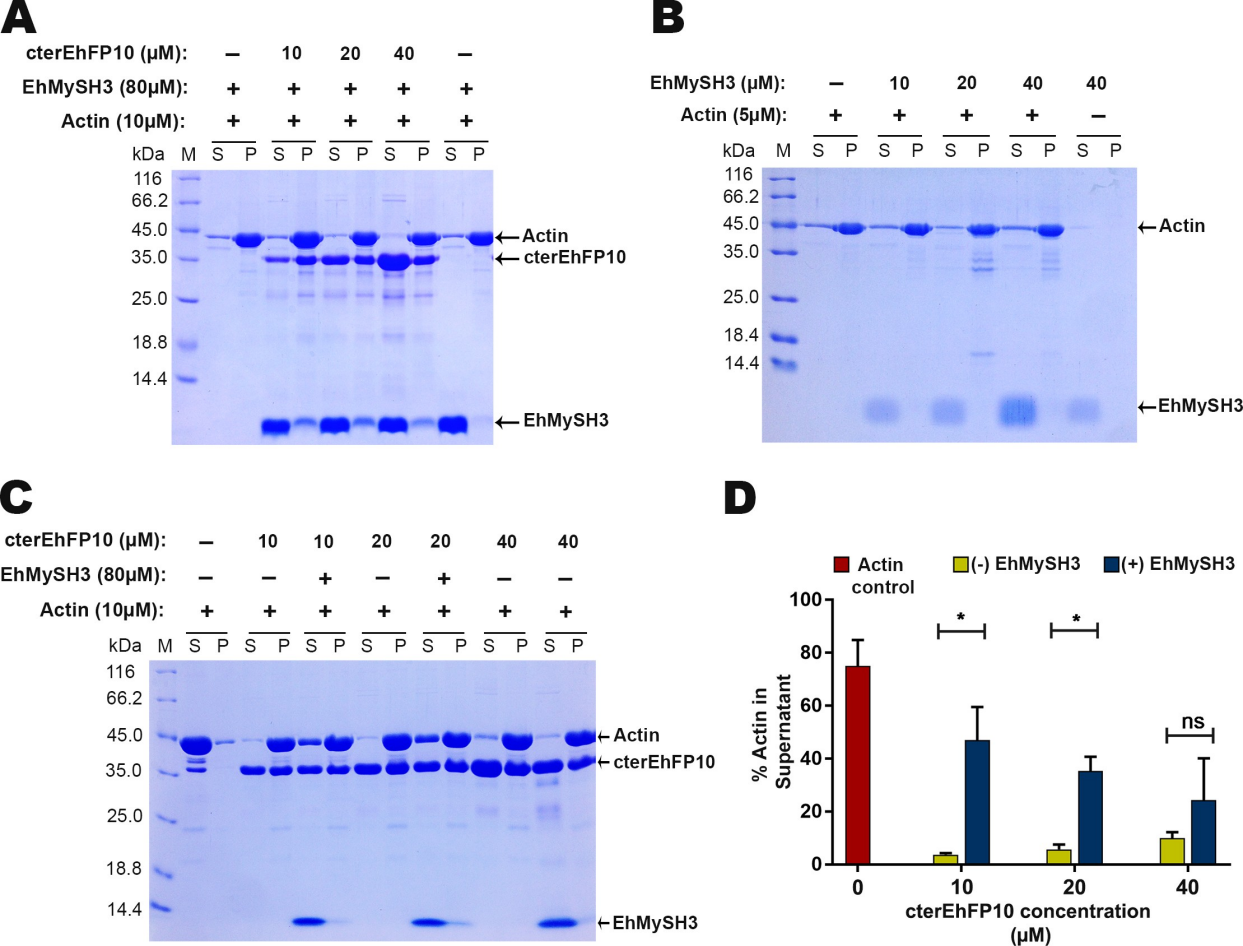
SH3 domain of *Eh*Myosin IB interacts with c-terminal domain of *Eh*FP10 and inhibits its actin bundling activity. (A) 15% SDS PAGE showing actin co-sedimentation assay with *Eh*MySH3 in the presence of cter*Eh*FP10. Actin filament was observed in the pellet fraction in all lanes. cter*Eh*FP10 was seen in the supernatant as well as in the pellet fraction with actin at all concentrations. *Eh*MySH3 was present in the supernatant fraction in the absence of cter*Eh*FP10. (B) 15% SDS PAGE gel showing the presence of *Eh*MySH3 in the supernatant fraction during actin co-sedimentation assay. This result indicated that *Eh*MySH3 does not bind to filamentous actin. (C) 12% SDS PAGE showing competitive inhibition of cter*Eh*FP10 mediated actin bundling activity by *Eh*MySh3. In the presence of the *Eh*MySH3 domain, a fraction of the actin filament remained in the supernatant, i.e., could not form bundles. As the concentration of cter*Eh*FP10 was increased, the amount of actin in the supernatant fraction decreased. (D) A bar diagram of the percentages of actin that remained in the supernatant samples, i.e., that could not form actin bundles, in the presence and absence of the *Eh*MySH3. Densitometry analysis was done by image J software. Statistical analysis was performed using unpaired two tailed Student’s t-test (n = 3, *Pvalue < 0.05). Error bars = SEM.

Our data clearly showed *Eh*FP10 to interact through its c-terminal domain with both F-actin and *Eh*Myosin IB. It is likely that the nature of the interaction between these three proteins could either be competitive or cooperative. To test our hypothesis, actin binding and bundling assays were performed using a constant amount of *Eh*MySH3 and various amounts of *Eh*FP10. *Eh*MySH3 by itself did not show any binding to rabbit muscle filamentous actin in a centrifugation-based actin co-sedimentation assay. *Eh*MySH3 was seen in the supernatant fractions while the filamentous actin was present in the pellet fraction (Fig 8B). As expected, association of *Eh*FP10 with *Eh*MySH3, led to some amount of the *Eh*MySH3 to come along with the pellet fraction (Fig 8A). The actin bundling assays were performed in the presence and absence of *Eh*MySH3 at a constant cter*Eh*FP10 concentration. Here, a greater percentage of actin was observed to be present in the supernatant fraction in the presence of *Eh*MySH3 than in its absence. This result suggested that the interaction of *Eh*Myosin IB with *Eh*FP10 to be competitive in nature, and the presence of myosin IB to inhibit the actin-bundling activity by *Eh*FP10. As the concentration of *Eh*FP10 was increased, it was able to overcome the inhibitory effect of *Eh*MySH3, as the majority of the actin filaments were seen in the pellet fraction (Fig 8C and 8D).

## Discussion

Processes such as phagocytosis, macropinocytosis and cell movement are highly dependent on actin filaments for extension of membrane protrusions. Several myosins such as myosin VI, myosin V and myosin I have been found to be associated with the endocytic processes, actin assembly and maintenance of cortical tension [36–38]. Unconventional myosin I plays a significant role during endocytic processes—directly by interacting with actin, and indirectly by recruiting factors associated with nucleation, polymerization, and stabilization of actin filaments [39]. The SH3 domain located in the tail region of myosin I plays a prominent role in bridging the processes of actin assembly and endocytosis.

The SH3 domain of myosin I isoforms in yeast interact with proline-rich regions of Vrp1 and recruits the Arp2/3 complex [40]. In amoebae, *Dictyostelium* myosin I recruits CARMIL via its SH3 domain and Acanthamoeba myosin I recruits Acan125. Both these proteins, regulate Arp2/3 mediated actin dynamics [41, 42]. Thus, in the present work, we set out to identify interacting partner(s) of the c-terminal SH3 domain of *E*. *histolytica* myosin IB, the only unconventinal myosin in this organism, to gain insight into the function of this important motor protein. On the basis of the crystal structure of *Eh*MySH3 [17] and predictions by SH3 HUNTER, a FYVE domain containing guanine nucleotide exchange factor (GEF), *Eh*FP10 was predicted to possess multiple proline-rich motifs in its c-terminal domain, which could interact with the yeast myosin I SH3 domain. The known targets of the myosin I SH3 domain have all been shown to be multi-domain proteins with PRDs (proline rich domains) and to help in the formation of the Arp2/3-mediated endocytic complex. These observations led us to further investigate the potential for *Eh*FP10 to serve as a binding partner of the *Eh*Myosin IB SH3 domain. SPR studies and the co-crystal structure of peptide P2 from cter*Eh*FP10 with the *Eh*MySH3 domain, as well as a pull-down assay validated the proposed interaction.

Cter*Eh*FP10 has a unique domain organisation, not present in any homologous GEFs. It has sequence similarity only with the APC basic domain from adenomatous polyposis coli protein and the tau protein domain, though it is very low. Both the APC basic and tau domains have been shown to bind actin filaments as well as microtubules and to each act as a molecular linker mediating actin-microtubule crosstalk [43, 44]. Tau has also been shown to bind SH3 domains like Fyn and cSRC and to facilitate cSRC-mediated actin rearrangements in neuronal cells [45, 46]. The *Eh*FP10 c-terminal domain showed biochemical similarity with the APC basic domain and tau, as it was seen to interact with filamentous actin and form thick bundled actin filaments. Interaction of *Eh*MySH3 with cter*Eh*FP10 was observed to inhibit the actin bundling activity of *Eh*FP10. Increasing the concentration of cter*Eh*FP10 was observed to overcome the inhibition and to result in an efficient bundling of the actin filaments.

*Eh*FP10 was found to be localized in the membrane ruffles and protrusion at the site of pinocytosis and phagocytosis in normal proliferating trophozoites and in cells induced for erythrophagocytosis, respectively. *Eh*FP10 was present from initiation until the closure of the cup during both phagocytosis and macropinocytosis. However, *Eh*FP10 was associated with the membrane encircling the newly internalized pinosomes, but disappeared after a while and was not present in newly formed phagosomes. This interesting observation states that there are differences in the regulation of these two endocytic processes. Previous studies have shown membrane ruffles to be regions of active actin polymerization and reorganization and these ruffles to eventually lead to the formation of macropinosomes. Macropinocytosis, like phagocytosis, is a process driven by the actin cytoskeleton. Formation and deformation of thick bundled actin filaments provide the force to push the membrane invagination and cell components inward, against the turgor pressure of the cell, apparently assisting in the endocytic cup progression. *Eh*Myosin IB was found to colocalize with *Eh*FP10 during macropinocytosis and phagocytosis in immunostained cells. *Eh*Myosin IB mostly localized to the tip of the progressing cup while *Eh*FP10 colocalized with both actin encircling the cup, and with *Eh*Myosin IB.

During Fc-receptor-mediated phagocytosis and macropinocytosis in macrophages, human Myo Ie (also known as myosin IC), a homolog of *Eh*Myosin IB, was shown to localize at the distal margin of the phagocytic cup and is believed to mediate a PI3K-dependent contractile activity for the closure of the cup aperture into an intra-cytoplasmic phagosome [47]. We hypothesize that in *Entamoeba*, *Eh*Myosin IB and *Eh*FP10 together are also involved in a similar kind of PI-3k-dependent contractile activity for endosome closure during endocytosis. As *Eh*FP10 contains an FYVE domain, known to recognize phosphoinositide-3-phosphate (PI-3P) formed by the action of PI-3 kinases. Hence, *Eh*Myosin IB and *Eh*FP10 must act downstream of PI-3 kinases. Also, *Eh*FP10 must be among the first molecules to be recruited to the endocytic cup as we showed in the live cell imaging results that it gets recruited to the membrane ruffles at an early stage, leading to endocytic cup formation. We hypothesize that *Eh*FP10 reorganizes the actin filaments into thicker bundles, which act as a driving force for membrane invagination and cup formation. As the cup advances to closure, *Eh*Myosin IB enriches into the tip of the growing cups; it interacts more with *Eh*FP10, which in turn inhibits actin bundling and promotes crosslinking of actin filaments, providing the required contractile activity leading to cup closure. However, further studies of myosin IB and *Eh*FP10 along with other regulatory proteins like Arp2/3, CaBP3, CaBP5, Rho GTPases, etc. remain to be carried out to obtain a complete overview of the pathway involved in the endocytic process.

The known effectors of the SH3 domain of unconventional myosin I have been found to be WASP and WASP-related proteins which regulate the actin dynamics directly or indirectly via the Arp2/3 complex leading to actin nucleation. Several GTPases act upon these effectors and adaptor proteins like Vrp1, Bee1p, Las17p, CARMIL, and Acan125 and this action leads to Arp2/3 dependent actin polymerization at the site of cup formation [39]. *E*. *histolytica* genome lacks the WASP/SCAR proteins but has WASP related proteins like WASH and MIM that possess VCA domain responsible for activating Arp2/3 complex mediated actin nucleation [48]. The ability of WASH and MIM proteins to promote actin nucleation and their involvement in phagocytosis still needs experimental validation [48, 49]. As *E*. *histolytica* lacks homologue of WASP/WAVE which links phosphoinositides mediated rho pathway and actin nucleation, the existence of a unique regulatory pathway which connects both is possible. Hence, this highly expressing, unique RhoGEF *Eh*FP10, which can regulate actin dynamics via its c-terminal tail and possibly with downstream RhoGTPases, is of great importance and advantage to the pathogen. Our findings have revealed a novel mode of regulation of endocytic processes in highly motile phagocytosing gastric pathogens such as *E*. *histolytica*.

## Materials and methods

### Cloning of various constructs

The primer sequences of *Eh*My1TD, GSTSH3, *Eh*GEFD, cter*Eh*FP10 and NGFP-*Eh*FP10 and their respective vector backbone and restriction site details are given in S1 Table. All of the genes were amplified by carrying out PCR (Eppendorf) using respective forward and reverse primers from the genomic DNA of *Entamoeba histolytica* strain HM1:IMSS. The PCR products and the vectors were double digested with their respective restriction enzymes (Thermo Fisher). These digested and purified products were then ligated using T4DNA ligase (Thermo Fisher) and kept at 16°C for 16 hours. The ligation mixture was then transformed into *E*.*Coli* DH5α, plated on antibiotic-containing LB agar plates and kept at 37°C overnight. The colonies were screened for positive clones. The clone was further confirmed by double digestion of isolated plasmids and gene sequencing.

### Protein overexpression and purification

The compositions of all the buffers used in purification are described in S2 Table. *Eh*MySH3 was purified as previously reported [17]. *Eh*My1TD, GSTSH3, and *Eh*GEFD recombinant plasmids were transformed into BL21 (DE3) and cter*Eh*FP10 in the *E*. *coli* strain BL21 (C41). The secondary culture was grown at 37°C using 1% primary culture grown overnight from a single BL21 colony and was induced with 0.2 mM IPTG for 4 hours. Pellets were resuspended in their respective lysis buffer and sonicated following 4–5 freeze-thaw cycles. After sonication, cell lysate was centrifuged at 13,000 rpm for 30 minutes. The supernatant obtained was loaded onto a Ni-NTA column (Sigma) with Ni-sepharose (GE healthcare). *Eh*My1TD, GSTSH3, and *Eh*GEFD proteins were eluted with their respective elution buffers following wash buffers. cter*Eh*FP10 protein was co-expressed with a chaperone. To remove the chaperone, after the protein was bound to Ni-sepharose, it was incubated for 1 hour in incubation buffer (2 mM Mg-ATP, 50 mM Tris pH-7.5, 300 mM NaCl, 10 mM MgCl_2_, 5% glycerol, 10 mM imidazole and 5 mM β-ME) and then was washed with a small amount of heat-denatured *E*. *coli* protein in the wash buffer. After 3–4 cycles of incubation followed by washes, cter*Eh*FP10 protein was eluted in the elution buffer. Purified proteins were concentrated using centricon filter (Amicon, Millipore) and further purified by carrying out gel permeation chromatography using a Superdex G75 16/60 column and G200 10/300 columns (GE Healthcare) pre-equilibrated in the buffer. The peak fractions were pooled together and checked using SDS-PAGE (S1 Fig). cter*Eh*FP10 showed a regular degradation pattern soon after removal of the chaperone; hence concentrated protein was frozen immediately after purification and stored at -80°C.

### Identification of *Eh*MySH3-binding peptides

Most SH3-domain-binding sequences can be classified into three categories: PxxP type, class I type [(K/R)xxPxxP] and class II type [xPxxPx(K/R)]. To identify proteins that interact with the c-terminal SH3 domain of *Eh*Myosin IB, a previously published proteomics data was utilized [30]. All of the listed proteins were screened for the presence of any of the three above-mentioned polyproline sequences by using the SH3 HUNTER web server [50]. From all the screened proteins, those having polyproline sequences similar to the ones recognised by yeast Myo3 and Myo5 (unconventional myosins) were shortlisted for further studies. Out of these, five peptides were selected for further studies, such that we had atleast one from each class of the SH3-binding polyproline sequences (Tables 1 and 2).

### Crystallization and data collection

For co-crystallization with all five synthesized peptides, *Eh*MySH3 protein samples, each at a concentration of 30 mg/ml, were mixed with the respective peptides in different molar ratios and incubated overnight at 4°C. Crystallization trials were done with several commercial screens from Hampton Research and Molecular Dimensions. After four days at 4°C, small needle-like crystals appeared for the *Eh*MySH3-P2 complex (mixed with in a molar ratio of 1:2) in crystal screen II (Hampton Research) conditions 0.2 M ammonium sulphate, 30% PEG 8000 and 0.2 M ammonium sulphate, 30% PEG 4000. Better crystals were obtained by macroseeding in 0.2 M ammonium sulphate and 30% PEG 8000 condition (S2 Fig). Diffractable crystals were flash frozen using mother liquor as a cryoprotectant in the cold room since the crystals were very temperature sensitive. The X-ray diffraction data for *Eh*MySH3-P2 were collected at the BM14 synchrotron beamline (ESRF, Grenoble, France). The data sets were indexed and scaled using HKL2000 [51].

### Structure determination

The structure was determined by molecular replacement using the PHASER MR Program of the CCP4 program suite [52, 53] with the crystal structure of the c-terminal SH3 domain of myosin IB from *E*. *histolytica* (PDB ID 5XGG) [17] as a template for the *Eh*MySH3-P2 peptide crystals. After a few cycles of manual building using COOT [54], refinement was done using REFMAC5 [55] in the CCP4 suite followed by PHENIX refine [56], resulting in a model with an R-factor of 0.19 (and R_free_ of 0.23. Data statistics are given in Table 3.

**Table 3:** Data collection and refinement statistics for the EhMySH3-P2 complex. Values given in brackets are for the higher-resolution shell.

Crystallographic Data	*Eh*MySH3-P2 complex
**PDB ID**	6A9C
**X-ray source**	ERSF BEAMLINE BM14
**Space group**	P2_1_2_1_2_1_
**Wavelength (**Å**)**	0.95
**Unit Cell Parameters**	
**a,b,c (**Å**)**	29.00, 60.0, 95.3
α, β, γ (°)	90, 90, 90
**Resolution range (**Å**)**	50.0–1.98 (2.01–1.98)
**R**_**sym**_ **or R**_**merge**_ **(%)**	0.10 (0.66)
**CC1/2**	0.9
**Completeness (%)**	99.7 (98.4)
**Redundancy**	14 (12.3)
**Mosaicity**	0.3
**Average I/**σ**(I)**	27.7 (3.3)
**No. of molecules in asymmetric unit**	2
**Refinement Statistics**	
**Resolution range (A°)**	37.32–1.98 (2.05–1.98)
**Total no. of observations**	109431 (10854)
**No. of unique observations**	12139 (1153)
**R**_**work**_	0.19 (0.23)
**R**_**free**_	0.21 (0.25)
**Mean B factor (A**°^**2**^**)**	32.04
**No. of atoms**	Protein 1099/ Water 51/ ligand 15
**RMSD Deviations**	
**Bonds (A°)**	0.02
**Bond angles (°)**	1.97
**Dihedral angles (°)**	20.3
**Cross validation error**	0.14
**Ramachandran Statistics**	
**Most-favoured region (%)**	91.7
**Allowed region (%)**	7.3
**Generously allowed region (%)**	0.6
**Disallowed region (%)**	0

#### Binding studies using surface plasmon resonance (SPR)

For binding studies of *Eh*MySH3 with peptides, an Autolab SPR apparatus was used at the Advanced Instrumentation Research Facility, Jawaharlal Nehru University, New Delhi, India. Here, the 11-mercaptoundecanoic acid (11MUA) monolayer on the surface of the gold chip (Autolab) was activated by *N*-hydroxysuccinimide (NHS; 0.05 M)/*N*-ethyl-*N*-(diethyl aminopropyl) and carbodiimide (EDC; 0.2 M). *Eh*MySH3 protein (ligand) at a concentration of 37 μM was immobilized in channel 1 on the activated surface of the gold chip in 10 mM sodium acetate buffer (pH 5.0) up to 700 RU. During experimentation, channel 1 was used to pass the analytes (peptides) while channel 2 was used as a blank (the signals of the analyte with a ligand-free surface). After ligand immobilization, the surface was blocked with 100 mM ethanolamine at pH 8.5, followed by regeneration using 50 mM NaOH. The different peptides, each at various concentrations, were each injected at a rate of 20 μl/min across the sensor chip surface and the association kinetics and dissociation kinetics were monitored for 200 s and 100 s, respectively. All of the dilutions were done in running buffer, i.e., 10 mM HEPES, 150 mM NaCl, 3 mM EDTA, 0.05% NP-40 surfactant [pH 7.4].

For interaction studies with the purified cter*Eh*FP10 domain, 1.175 ng/mm^3^ of *Eh*MySH3 was immobilized on the activated chip surface and samples of the cter*Eh*FP10 domain having different concentrations were passed over it. Association kinetics was studied for 400 s while the dissociation was monitored for 150 s. 1M NaCl was used for regeneration of the chip surface. SPR studies with F-actin was carried out by immobilizing 0.67 ng/mm^3^ of filamentous actin on the chip surface using the buffer 50 mM sodium acetate pH 4.1, 0.05 mM CaCl_2_, 0.05 mM ATP and 0.125 mM DTT. (A solution of 0.5 mg/ml F-actin was obtained by dissolving actin powder (Sigma) in 50 mM sodium acetate pH 4.0, 12.5 mM KCl, 0.5 mM MgCl_2_ and 0.25 mM ATP).

All of the data were recorded at 25°C. For the evaluation of the kinetics, differential sensorgrams were analyzed using Autolab SPR Kinetic Evaluation software.

### GST Pull-down assay

Purified GSTSH3 and GST were incubated for one hour at 4°C with glutathione Sepharose (GE Healthcare Lifesciences) pre-equilibrated in equilibration buffer (50 mM Tris pH 7.4, 150 mM NaCl). Then, it was washed thrice with the equilibration buffer and incubated with the same amount of purified cter*Eh*FP10 for two hours at 4°C. Following 3–4 washes with wash buffer (50 mM Tris pH 7.4, 300 mM NaCl), the proteins were eluted with 20 mM reduced glutathione, 50 mM Tris pH 7.4, and 150 mM NaCl. The fractions were loaded onto a 12% SDS-PAGE gel and analyzed.

### Actin co-sedimentation and F-actin bundling assay

G-actin was purified from rabbit muscle powder (sigma) [57]. For F-actin preparation, purified G-actin at a concentration of 23 μM was dissolved in G-buffer (20 mM Tris-Cl, 0.5 mM DTT, 0.2 mM ATP, 0.1 mM CaCl_2_and 0.1 mM NaN_3_) and was allowed to polymerize for 1½ hours at RT by adding to its solution an I_mix_ solution (50 mM KCl, 2 mM MgCl_2_, and 0.5 mM ATP). cter*Eh*FP10 and *Eh*MySH3 proteins of various concentrations were each added to separate samples of the mixture containing polymerized F-actin in a total volume of 200 μl and incubated for 30 minutes at RT. Each of the resulting mixtures was centrifuged at 40,000 rpm for two hours in a Beckman ultracentrifuge. Pellet and supernatant fractions were collected and analyzed using 10% SDS-PAGE followed by Coomassie blue staining.

For bundling assays, cter*Eh*FP10 and *Eh*MySH3 proteins of various concentrations were each added to separate samples of the mixture containing polymerized F-actin in a total volume of 50 μl and incubated for 30 minutes at RT. Each mixture was centrifuged at 12,000 rpm for 10 minutes. Pellet and supernatant fractions were collected and analyzed using 10% SDS-PAGE followed by Coomassie blue staining.

Purified alpha-actinin from rabbit skeletal muscle (cytoskeleton Inc.) was used as positive control and Bovine serum albumin (SRL) was used as negative control for both binding and bundling studies.

### Antibody generation

The purified antigenic proteins (*Eh*My1TD for m*Eh*Myosin1B and *Eh*GEFD for rEhFP10 Ab) were dialyzed against PBS. Six mice were immunized subcutaneously with 100 μg of protein per mice per injection with an interval of two weeks between each injection. The first dose of the protein was emulsified with complete Freund’s adjuvant while the following doses were emulsified with incomplete Freund’s adjuvant. Following the immunization series, the serum of each mouse was stored in aliquots at -80°C. To check the titer of the polyclonal antibody, western analysis of the *E*. *histolytica* lysate was performed.

### *E*. *histolytica* culture maintenance and transfection

*E*. *histolytica* strain HM1:IMSS trophozoites were grown axenically in TYI-S-33 medium [58]. The cells were maintained and grown in TYI-33 medium complemented with 15% adult bovine serum, 1X Diamond’s vitamin mix and antibiotic (125 μl of 250 U/ml benzyl penicillin and 0.25 mg/ml streptomycin per 90 ml of medium). Transfection was performed by carrying out electroporation as described previously [59]. Briefly, trophozoites in log phase were harvested and washed with PBS followed by incomplete cytomix buffer [10 mM K_2_HPO_4_/KH_2_PO_4_ (pH 7.6), 120 mM KCl, 0.15 mM CaCl_2_, 25 mM HEPES (pH 7.4), 2 mM EGTA, 5 mM MgCl_2_]. The washed cells were then resuspended in 0.8 ml of complete cytomix buffer (incomplete cytomix containing 4 mM adenosine triphosphate, 10 mM glutathione) containing 200 μg of plasmid DNA and subjected to two consecutive pulses of 3000 V/cm (1.2 kV) at 25 μF (Bio-Rad, electroporator). The transfectants were initially allowed to grow without any selection. Drug selection was initiated after two days of transfection in the presence of 10 μg/ml G-418 for constructs with luciferase reporter gene.

### Preparation of *E*. *histolytica* cell lysate

One million trophozoites growing in log phase were harvested by centrifugation at 280 g for 7 min at 4°C. The pellet was then washed with chilled PBS pH 7.2 and pelleted. For probing *Eh*FP10, washed cells were fixed with 3.7% pre-warmed paraformaldehyde for 60 min at room temperature. After the cells were fixed, they were again harvested at 280 xg for 7 min. In each of the two cases, the pellet was heated at 80°C for one minute, and then resuspended in 2x SDS dye without β-ME. The lysate was heated at 100°C for 7 minutes and centrifuged at 13000 rpm for 15 minutes to pellet down the debris. The supernatant was aliquoted and stored, and quantification was achieved by performing a BCA.

### Immunofluorescence staining

*E*. *histolytica* trophozoites and transfectants were resuspended in incomplete TY1-33 medium and transferred onto acetone-cleaned coverslips placed in a Petri dish. The cells were allowed to adhere for 5 min at 37°C. The culture medium was discarded and the cells were fixed with 3.7% pre-warmed paraformaldehyde for 30 min. After fixation, the cells were permeabilized with 0.1% Triton X-100/PBS for 5 min, washed with PBS and then quenched for 30 min in PBS containing 50 mM NH_4_Cl. The coverslips were blocked with 1% BSA/PBS for 2 h, followed by incubation with primary antibody at 37°C for 1.5 h. The coverslips were washed three times with 1% BSA/PBS and then incubated with secondary antibody for 45 min at 37°C. Antibody dilutions used were m*Eh*Myo1B at 1:100, anti-GFP (Sigma) at 1:100, rEhFP10 Ab at 1:150, anti-rabbit/mice Alexa 488, Alexa 556 and Pacific blue-410 (Molecular Probes) at 1:250, TRITC-Phalloidin at 1:250. Cells were further washed with 1% BSA/PBS twice and then PBS once and mounted on a glass slide using 1, 4-diazbicyclo (2,2,2) octane (DABCO) (55) 2.5% in 80% glycerol. The edges of the coverslip were sealed with nail-paint to avoid their becoming dried out. Confocal images were made using an Olympus Fluoview FV1000 laser scanning microscope.

### Time-lapse imaging

Amoebic cells expressing *Eh*FP10 tagged at its N-terminus with GFP, (NGFP-*Eh*FP10) were plated onto a 35 mm glass bottom dish and allowed to adhere to the dish. RBCs were labelled using CFDA SE dye (Carboxyfluorescein diacetate succinimidyl ester, Thermo Fisher Scientific). RBCs were collected by pricking a finger with a needle and collecting the blood in PBS. The cells were washed with PBS twice followed by incubation in PBS containing 10 μM CFDA for 10 min at 37°C with intermittent tapping. The reaction was stopped by washing the RBCs with PBS and then the RBCs were kept in ice. Labeled RBC or TRICT-Dextran (Sigma) containing media was added and time-lapse imaging was done using a spinning disk confocal microscope (Nikon A1R, Optics- Plan Apo VC606 oil DIC N2, Camera- Nikon A1, NA-1.4, RI-1.515). The temperature was maintained at 37°C with the help of a chamber provided along with the microscope. The images were captured at 500 ms intervals. The raw images were processed using NIS element 3.20 analysis software.

### Statistical analysis

All of the statistical analyses were done using GraphPad PRISMA 7 software using Student’s two-tailed t-test. Images of cells which were immunostained for all three proteins *Eh*FP10, myosin IB and actin proteins were used for quantitation. Data with P < 0.05 were considered to be significant.

## Supporting information

S1 Video Localisation of EhFP10 in *E*. *histolytica* cells. Live cell movie showing localisation of GFP tagged EhFP10 protein (green) within *E*. *histolytica* cells.
(AVI)

S2 Video Involvement of EhFP10 in phagocytosis. Live cell movie showing localisation of GFP tagged EhFP10 protein (green) within *E*. *histolytica* cells activated for phagocytosis. RBC are red in colour.
(AVI)

S3 Video Involvement of EhFP10 in pinocytosis. Live cell movie showing localisation of GFP tagged EhFP10 protein (green) within *E*. *histolytica* cells performing macropinocytosis. Media is red in colour. Engulfed media can be seen as red circular regions within the cell.
(AVI)

S1 Fig SDS-PAGE gel showing purified proteins. **(A)** *Eh*My1TD, **(B)** GSTSH3, **(C)** *Eh*GEFD, and **(D)** cter*Eh*FP10. **(E)** Intact mass analysis of cterEhFP10: MS spectrum depicting a major peak equivalent to 28 kDa i.e., the molecular weight of cterEhFP10 although its runs on 12% SDS-PAGE gel at around 35 kDa.
(TIF)

S2 Fig Crystals of the *Eh*MySH3-P2 complex. (A) Initial hits of *Eh*MySH3 were obtained in 30% PEG 8000, 0.2 M ammonium sulphate condition of Crystal Screen II (Hampton Research) at 4°C.
(B) Diffractable *Eh*MySH3 crystals were obtained after macroseeding with a few crystals from initial hits in the same condition.
(TIF)

S3 Fig *Eh*FP10 has a unique domain structure. A multiple-sequence alignment of EhFP10 sequences from various species of *Entamoeba* of *E*. *histolytica*. (*E*. *histolytica* and *E*. *nuttalli* are virulent, *E*. *dispar* is non-virulent and *E*. *invadens* causes infections in reptiles). Inspection of the alignment showed that the maximum differences could be seen in the c-terminal domain region involved in actin reorganisation. *E*. *dispar* is 95 percent identical to *E*. *histolytica* while *E*. *invadens* is only 35 percent.
(TIF)

S4 Fig (A) Images from immunofluorescence studies with anti-GFP antibody in GFP-EhFP10-overexpressed cells, and EhFP10-specific antibodies showing both to colocalize well.
(B) Bar graph depicting colocalization of GFP-tagged EhFP10 and untagged EhFP10. (C) Western blot depicting a band at 100 kDa equivalent to EhFP10 protein in wild type HM1 total lysate. Prebleed was used as a negative control. Ehcoactosin was used as a loading control. (D) Western blot depicting a band at about 130 kDa in lysate of GFP-EhFP10 cells while the GFP vector control showed only a band corresponding to GFP.
(D, E) Images from immunofluorescence studies in wild-type E. histolytica cells showed EhFP10 localized in membrane ruffles and cup-like projections and within pseudopod extensions and closing vesicles, during both pinocytosis and phagocytosis.
(TIF)

S1 Table Details of various clones used in the study. (DOCX)

S2 Table Details of protein expression and purification buffer composition. (DOCX)
