# Supplementary figures and images for: EhFP10: A FYVE family GEF interacts with myosin IB to regulate cytoskeletal dynamics during endocytosis in Entamoeba histolytica
Source: PLoS Pathog. 2019 Feb 19;15(2):e1007573. doi: 10.1371/journal.ppat.1007573 (PMC6396940; doi:10.1371/journal.ppat.1007573)

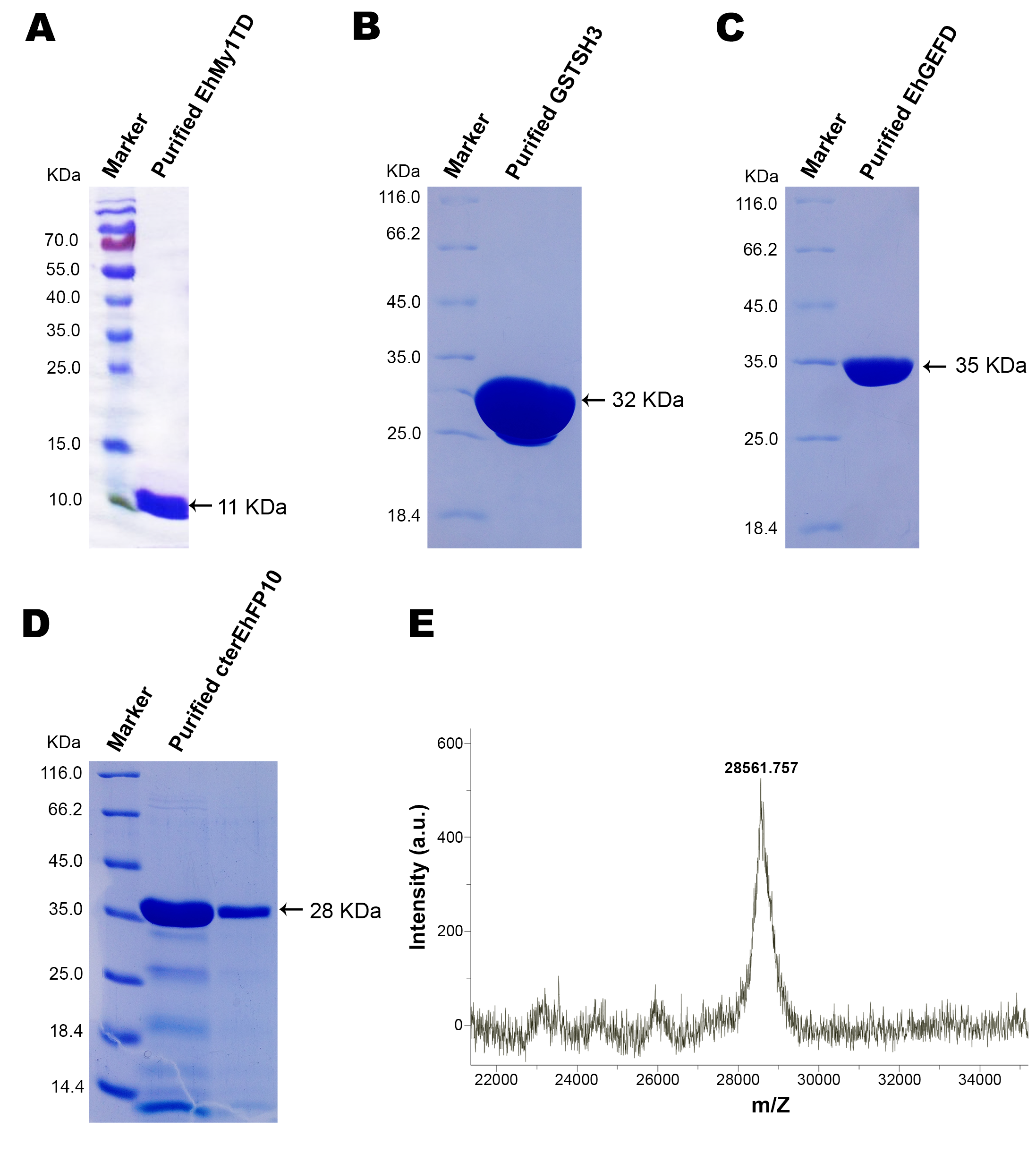

Supplement: S1 Fig — (A) EhMy1TD, (B) GSTSH3, (C) EhGEFD, and (D) cterEhFP10. (E) Intact mass analysis of cterEhFP10: MS spectrum depicting a major peak equivalent to 28 kDa i.e., the molecular weight of cterEhFP10 although its runs on 12% SDS-PAGE gel at around 35 kDa. (TIF) [file ppat.1007573.s004.tif]

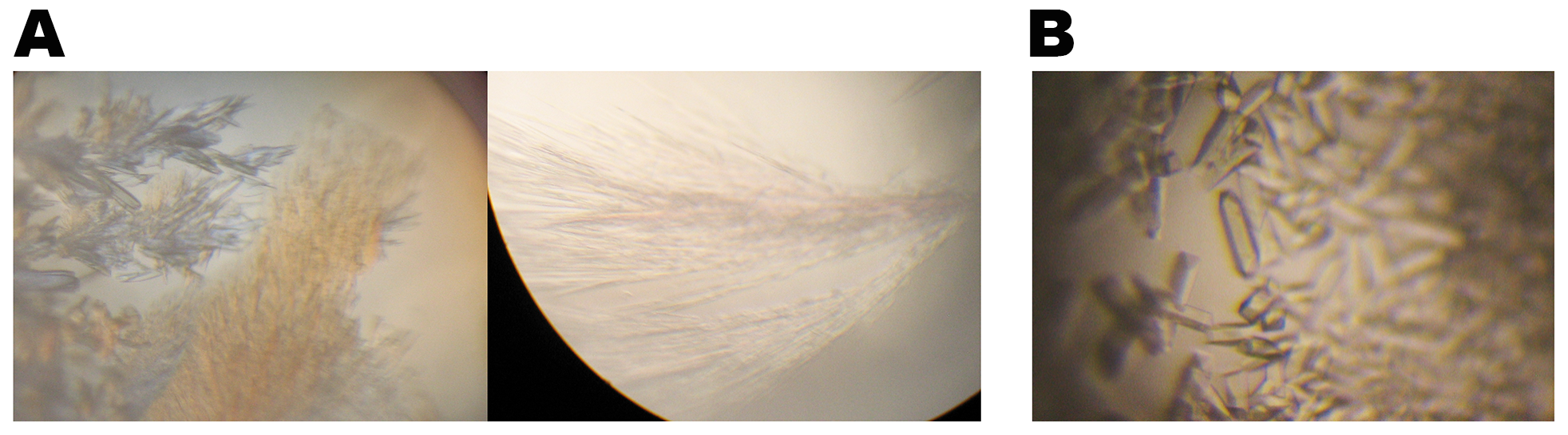

Supplement: S2 Fig — (A) Initial hits of EhMySH3 were obtained in 30% PEG 8000, 0.2 M ammonium sulphate condition of Crystal Screen II (Hampton Research) at 4°C. (B) Diffractable EhMySH3 crystals were obtained after macroseeding with a few crystals from initial hits in the same condition. (TIF) [file ppat.1007573.s005.tif]

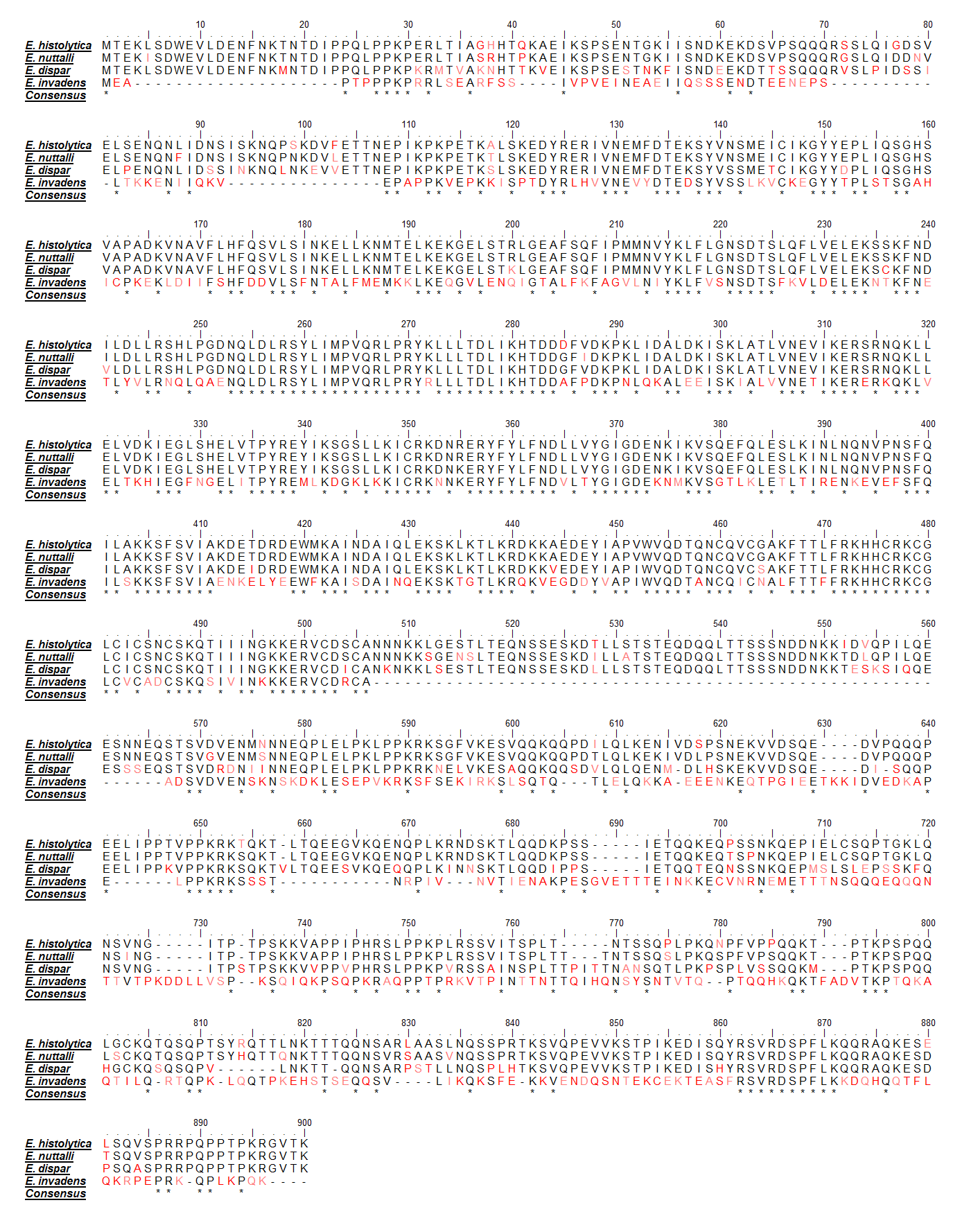

Supplement: S3 Fig — A multiple-sequence alignment of EhFP10 sequences from various species of Entamoeba of E. histolytica. (E. histolytica and E. nuttalli are virulent, E. dispar is non-virulent and E. invadens causes infections in reptiles). Inspection of the alignment showed that the maximum differences could be seen in the c-terminal domain region involved in actin reorganisation. E. dispar is 95 percent identical to E. histolytica while E. invadens is only 35 percent. (TIF) [file ppat.1007573.s006.tif]

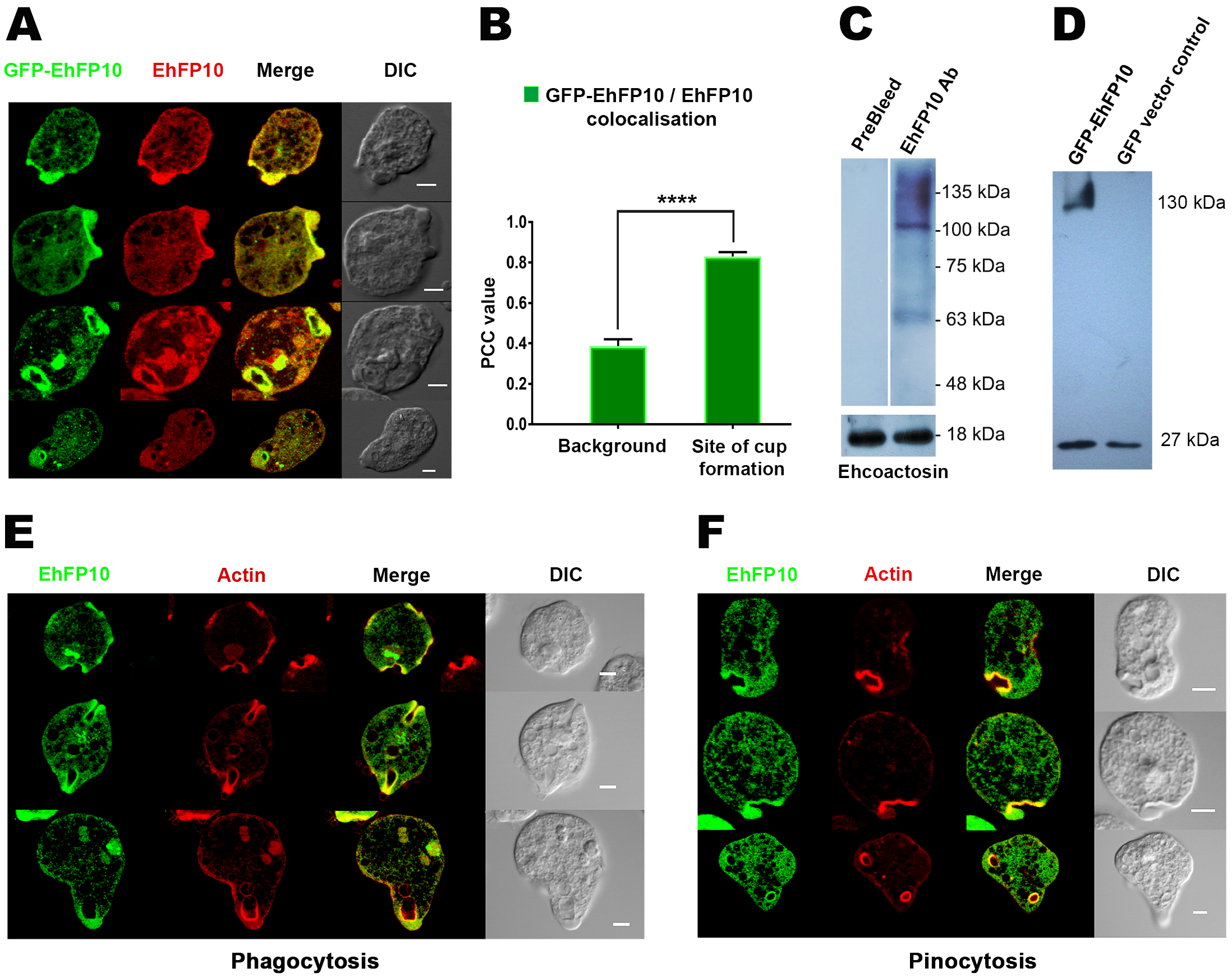

Supplement: S4 Fig — (A) Images from immunofluorescence studies with anti-GFP antibody in GFP-EhFP10-overexpressed cells, and EhFP10-specific antibodies showing both to colocalize well. (B) Bar graph depicting colocalization of GFP-tagged EhFP10 and untagged EhFP10. (C) Western blot depicting a band at 100 kDa equivalent to EhFP10 protein in wild type HM1 total lysate. Prebleed was used as a negative control. Ehcoactosin was used as a loading control. (D) Western blot depicting a band at about 130 kDa in lysate of GFP-EhFP10 cells while the GFP vector control showed only a band corresponding to GFP. (D, E) Images from immunofluorescence studies in wild-type E. histolytica cells showed EhFP10 localized in membrane ruffles and cup-like projections and within pseudopod extensions and closing vesicles, during both pinocytosis and phagocytosis. (TIF) [file ppat.1007573.s007.tif]
