# Supplementary material for: EhFP10: A FYVE family GEF interacts with myosin IB to regulate cytoskeletal dynamics during endocytosis in Entamoeba histolytica
Source: PLoS Pathog. 2019 Feb 19;15(2):e1007573. doi: 10.1371/journal.ppat.1007573 (PMC6396940; doi:10.1371/journal.ppat.1007573)
Supplement: S1 Table — (DOCX) [file ppat.1007573.s008.docx]

| S.No. | Construct name | Amino acid residue position  (start – end)  [ accession no.] | Primer sequence | Plasmid vector | Restriction enzyme site |
| --- | --- | --- | --- | --- | --- |
| 1. | *Eh*My1TD | 1045 – 1049  [[EAL48894](https://www.ncbi.nlm.nih.gov/protein/EAL48894)] | **FwMy1TD:**  5’-CGCCATATGTCTTCTGTTTCTTCTTATGCTGCTC-3’  **RvMy1TD :**  5’-CCGCTCGAGAATTTCTTTGACATAGTTGTTAGGAATC- 3’ | pET21c | NdeI - XhoI |
| 2. | GSTSH3 | 994 – 1049  [[EAL48894](https://www.ncbi.nlm.nih.gov/protein/EAL48894)] | **FwGSTSH3:**  5’-CCGGGGATCCCAAGTTAAAGCACTCTATCCATATACTG-3’  **RvGSTSH3:**  5’-CCCCTCGAGTTAAATTTCTTTGACATAGTTGTTAGGAA-3’ | pGEX-6P2 | BamHI - XhoI |
| 3. | *Eh*GEFD | 113 – 438  [EAL46050] | **FwGefDHPH:**  5’-CATGCCATGGCTAAACCTGAAACTAAAGCATTATC-3’  **RvGEFDHPH:**  5’-CCGCTCGAGTAAAGTTTTTAATTTTGACTTTTCC-3’ | pET28(b) | NcoI - XhoI |
| 4. | cterEhFP10 | 631- 876  [EAL46050] | **FwAPCGEF:**  5’-CATGCCATGGCTGTCCCTCAACAACAACCAGAA-3’  **RvGEFfull:**  5'-CCGCTCGAGTTTTGTAACCCCTCTTTTTGG 3' | pET28(b) | NcoI - XhoI |
| 5. | NGFP-EhFP10 | 1 – 876  [EAL46050] | **FwGFPNterGEF:**  5’-CCGCTCGAGATGACTGAAAAGCTAAGTGATTGG-3’  **RvGFPNterGEF:**  5’-CCGGGGATCCTTATTTTGTAACCCCTCTTTTTGG-3’ | p*Eh*-NEOGFP | XhoI - BamHI |
