# Supplementary material for: EhFP10: A FYVE family GEF interacts with myosin IB to regulate cytoskeletal dynamics during endocytosis in Entamoeba histolytica
Source: PLoS Pathog. 2019 Feb 19;15(2):e1007573. doi: 10.1371/journal.ppat.1007573 (PMC6396940; doi:10.1371/journal.ppat.1007573)
Supplement: S2 Table — (DOCX) [file ppat.1007573.s009.docx]

| **S.No.** | **Protein** | **Lysis Buffer** | **Wash Buffer** | **Elution Buffer** | **GPC Buffer** |
| --- | --- | --- | --- | --- | --- |
| 1. | GSTSH3 | 50 mM Tris pH-7.4  150 mM NaCl  5% glycerol  3 mM β-ME | 50 mM Tris pH7.4  300 mM NaCl  3mM β-ME | 50 mM Tris pH-7.4  150 mM NaCl  20 mM reduced glutathione  5% glycerol  3 mM β-ME | 50 mM Tris pH-7.4  150 mM NaCl  5% glycerol  3 mM β-ME |
| 2. | *Eh*My1TD | 50 mM Tris pH-7.4  150 mM NaCl | **Wash buffer1:**  50 mM Tris pH-7.4  **Wash buffer2:**  50 mM Tris pH7.4  300 mM NaCl  **Wash buffer3:**  50 mM Tris pH7.4  150 mM NaCl  20 mM imidazole | 50 mM Tris pH-7.4  150 mM NaCl  100 mM Imidazole | 50 mM Tris pH-7.4  150 mM NaCl |
| 3. | cter*Eh*FP10 | 50 mM Tris pH-7.4  150 mM NaCl  10 mM MgCl_2_  5% glycerol  10 mM imidazole  5 mM β-ME  0.1% Triton-X 100 | 50 mM Tris pH-7.4  300 mM NaCl  10 mM MgCl_2_  5% glycerol  10 mM imidazole  5 mM β-ME | 50 mM Tris pH-7.4  150 mM NaCl  100 mM Imidazole  5% glycerol  5 mM β-ME | 50 mM Tris pH-7.4  150 mM NaCl  5% glycerol  5 mM β-ME |
| 4. | *Eh*GEFD | 50 mM Tris pH-7.4  200 mM NaCl  5% glycerol  3 mM β-ME | 50 mM Tris pH-7.4  200 mM NaCl  5% glycerol  5 mM imidazole  3 mM β-ME | 50 mM Tris pH-7.4  200 mM NaCl  100 mM Imidazole  5% glycerol  3 mM β-ME | 30 mM Tris pH-7.4  200 mM NaCl  5% glycerol  3 mM β-ME |
